# Biomaterials mediated 3R (remove-remodel-repair) strategy: holistic management of *Helicobacter pylori* infection

**DOI:** 10.1186/s12951-025-03455-2

**Published:** 2025-07-01

**Authors:** Tinglin Zhang, Yating Zheng, Tielou Chen, Yuankai Gu, Yingli Gong, Dewei Wang, Zhaoshen Li, Yiqi Du, Li Zhang, Jie Gao

**Affiliations:** 1 Changhai Clinical Research Unit, Shanghai Changhai Hospital, Naval Medical University, Shanghai, 200433 China; 2 Shanghai Key Laboratory of Nautical Medicine and Translation of Drugs and Medical Devices, Shanghai, 200433 China; 3 Department of Gastroenterology, Shanghai Changhai Hospital, Naval Medical University, Shanghai, 200433 China; 4 Department of Stomatology, Shanghai Changhai Hospital, Naval Medical University, Shanghai, 200433 China; 5 Department of Neurology, Shanghai Changzheng Hospital, Naval Medical University, Shanghai, 200003 China; 6 College of Life Science, Mudanjiang Medical University, Mudanjiang, 157011 China; 7 Yangzhou Branch of Jiangsu Provincial Corps of Chinese People’s Armed Police Force, Jiangsu, 225007 China; 8 College of Science, University of Shanghai for Science and Technology, Shanghai, 200433 China

**Keywords:** *Helicobacter pylori*, Holistic integrative medicine, Multimodal therapy, Biomaterials, Alternative antibiotics

## Abstract

*Helicobacter pylori* (HP) is a major etiological agent of gastric cancer, with a global prevalence of around 50%. Current treatments, primarily based on antibiotics, face challenges such as increasing drug resistance and disruption of the gut microbiota. This review proposes a holistic integrative medicine (HIM) approach, guided by the 3R concept (Remove, Remodel, and Repair), to address these limitations. The 3R concept offers a novel paradigm for the integrated prevention and treatment of HP infections: Remove targets the direct eradication of HP by overcoming antibiotic resistance, Remodel focuses on reshaping the immune microenvironment to clear pathogens, and Repair emphasizes the restoration of the gastric mucosa and protection of the gut microbiota. We discuss the potential of biomaterials, including nanoparticles for targeted drug delivery and ROS generation, hydrogels for sustained release and mucosal repair, microspheres for enhanced drug loading and controlled release, and probiotics for microbiota restoration. Additionally, multimodal therapies such as phototherapy, sonodynamic therapy, and magnetic hyperthermia provide non-invasive, targeted treatments. These innovations align with HIM principles, integrating pathogen eradication with mucosal healing and microbiome protection. Future research should focus on optimizing these materials and validating their clinical applicability to improve patient outcomes and combat antibiotic resistance.

**Supplementary Information:**

The online version contains supplementary material available at 10.1186/s12951-025-03455-2.

## Introduction

**Fig. 1:**
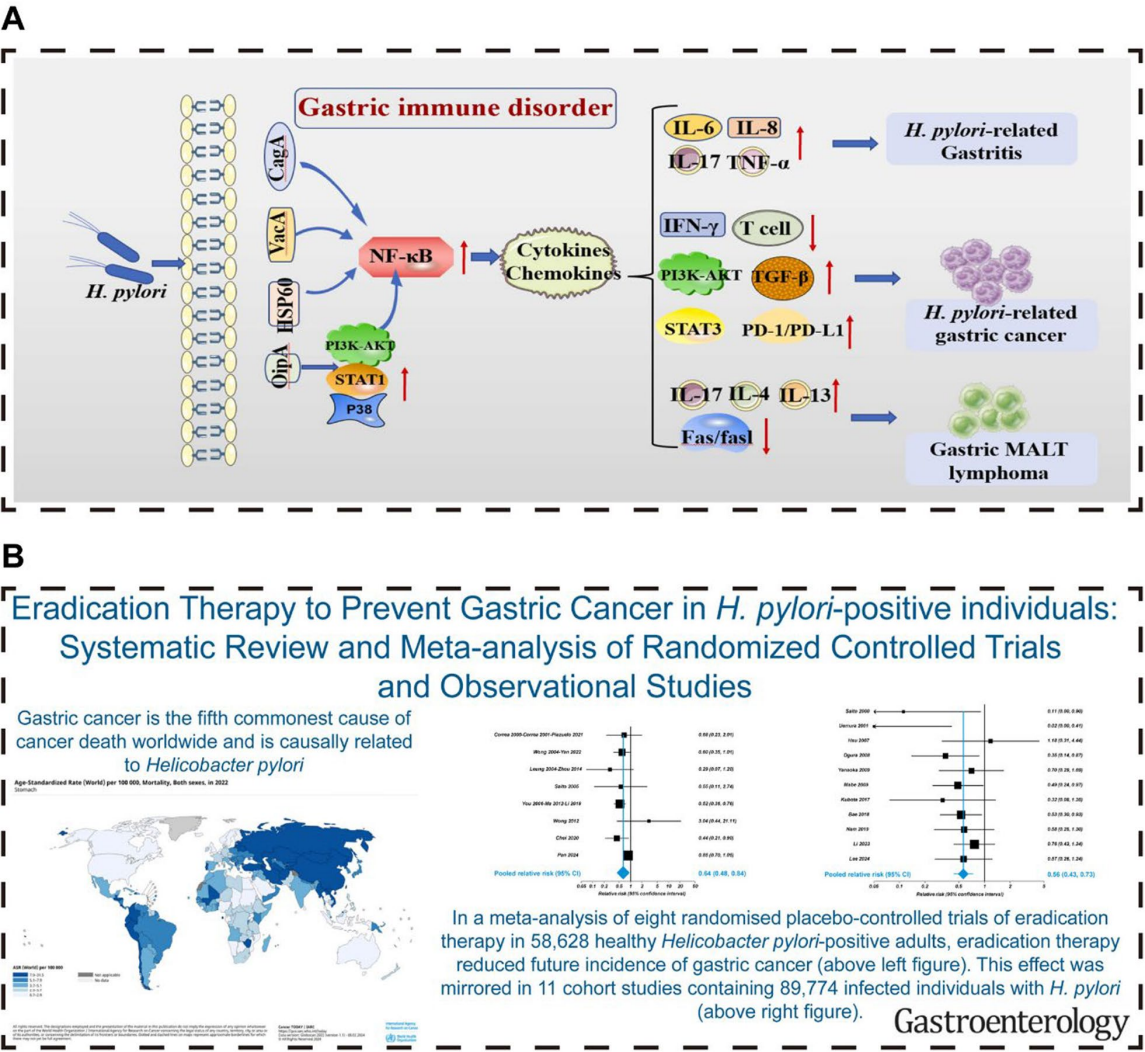
Relationship between HP and gastric cancer. **A** Mechanisms of HP-induced gastric cancer: HP infection disrupts the balance of the gastric mucosal immune system, thereby facilitating the development of HP-associated gastritis, gastric cancer, and gastric mucosa-associated lymphoid tissue (MALT) lymphoma. The diagram illustrates the complex interplay of immune factors and pathways, where upward arrows signify upregulation or activation, and downward arrows denote downregulation or inhibition of these immune responses. **B** Eradication of HP reduces the risk of gastric cancer. This meta-analysis concludes that HP eradication therapy significantly reduces the incidence of gastric cancer and gastric cancer-related mortality in HP-positive individuals. **A **Reproduced with permission from ref, [5] ©Liu S (2023). **B** Reproduced with permission from ref, [8] © Ford AC (2025)

Gastric cancer ranks as the fifth most prevalent malignant tumor globally, with high incidence and mortality rates that make it the third leading cause of cancer-related deaths [1]. The late-stage diagnosis of many patients contributes to the high mortality rate [2]. In 2018, gastric cancer resulted in a staggering 784,000 deaths worldwide. *Helicobacter pylori* (HP), a gram-negative bacterium residing in the gastric mucosa, is a significant risk factor for gastric cancer [3, 4]. Approximately 90% of gastric cancer cases are linked to HP infection (Fig. 1A) [5]. As an ancient pathogen, HP has coexisted with humans for approximately 60,000 years, infecting about half of the global population [6]. The 2015 Kyoto Global Consensus Report officially categorized HP infection as an infectious disease, highlighting its role in gastric cancer development and advocating treatment for all infected individuals [7]. Eradicating HP has been shown to significantly reduce the incidence and mortality of gastric cancer by 47% (Fig. 1B), respectively [8]. The decline in gastric cancer incidence is correlated with the decreasing global prevalence of HP, emphasizing the importance of HP eradication for gastric cancer prevention and gastric health improvement [9]. Consequently, the latest medical guidelines recommend treating individuals with HP infection to prevent gastric cancer [10].

The threat of HP infection and gastrointestinal diseases to human health is substantial, imposing a significant economic burden on healthcare systems [11]. Our research team, led by Academician Zhaoshen Li and Director Yiqi Du from the Digestive Disease Department and Clinical Research Center of Shanghai Changhai Hospital, Naval Medical University, has conducted extensive research in the field of HP [12–14]. We completed a large-scale, nationwide, family-based epidemiological survey of HP infection, revealing that the average individual and family infection rates in China are 40.66% and 71.21%, respectively [12]. This work established the first set of family-based guidelines for HP eradication [13]. A 2023 review in the journal Gut by Nobel laureate in Physiology or Medicine and HP Discoverer, Academician Marshall, supported our family-based screening and eradication of HP as a viable and commendable strategy [15]. Furthermore, a nationwide multicenter long-term follow-up study confirmed that eradicating HP can effectively lower the incidence of gastric cancer [16]. Therefore, eradicating HP is crucial for reducing the incidence of gastric cancer and safeguarding human health.

**Fig. 2:**
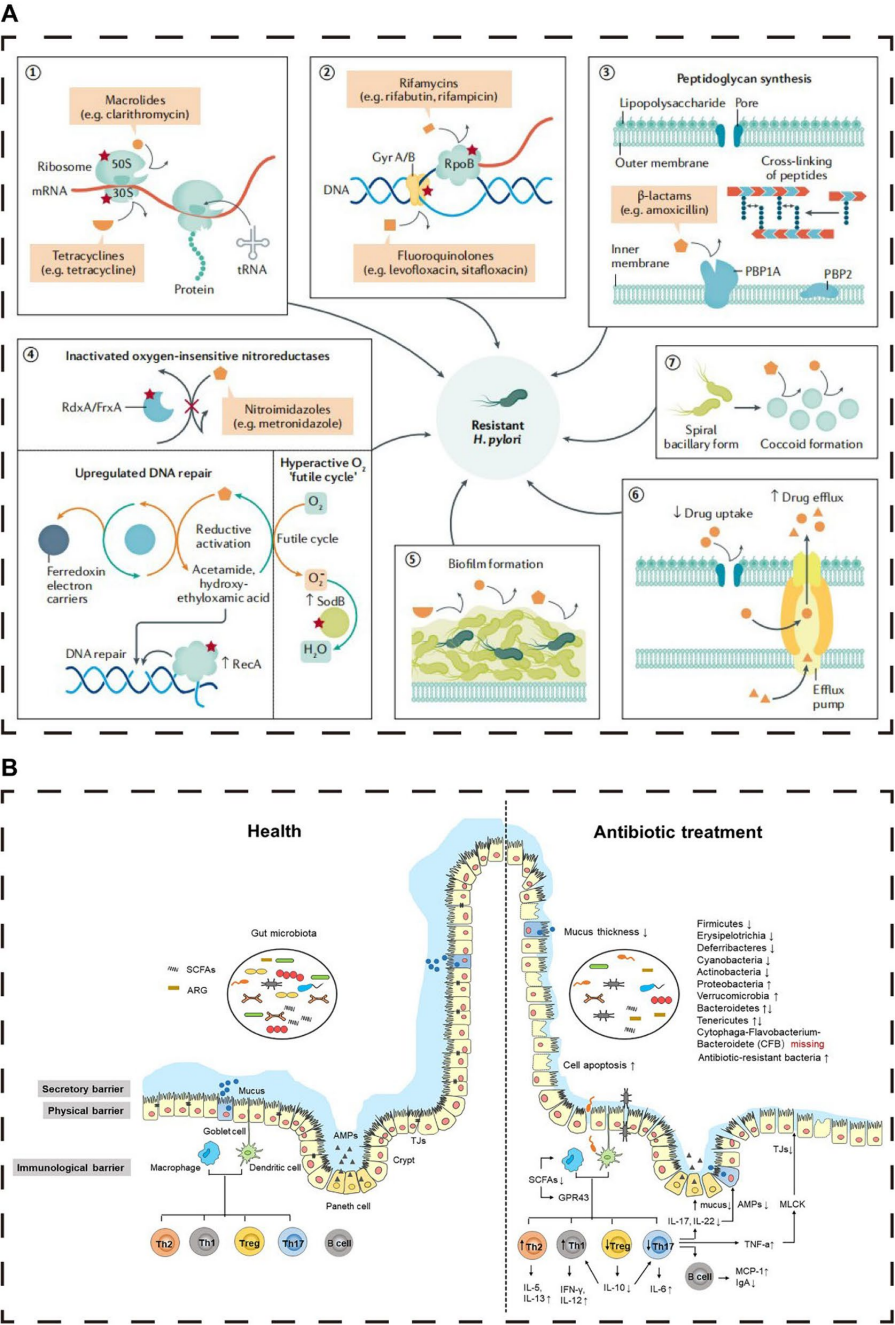
Biological characterization of HP resistance and the impact of antibiotics on intestinal microecology. **A** The diagram illustrates the biological underpinnings of drug resistance in HP, with red asterisks denoting sites prone to mutations that confer resistance. The primary mechanisms include the development of factors that impede antibiotic cellular penetration and molecular activity by modifying drug targets (1–3), the suppression of intracellular drug activation (4), biofilm formation, which acts as a barrier (5), the augmentation of drug efflux coupled with a decrease in drug absorption (6), and the induction of ultrastructural and metabolic shifts (7). These mechanisms are not mutually exclusive and can coexist across various strains, collectively contributing to three predominant patterns of resistance: SDR, MDR, and HR. **B** Diagram depicting the detrimental effects of antibiotics on the intestinal microecology. Antibiotic exposure can significantly alter the gut microbiota composition, precipitating a dysbiotic state and undermining intestinal barrier integrity. This disruption can affect the secretory, physical, and immunological components of the barrier, potentially leading to a range of health complications. **A** Reproduced with permission from ref, [19] © Tshibangu-Kabamba E (2021).** B** Reproduced with permission from ref,[20] © Duan H (2022).

The current therapeutic landscape for HP infection predominantly relies on multidrug regimens, encompassing antibiotics, proton pump inhibitors (PPIs), and bismuth compounds [10, 17, 18]. Traditional antibiotic treatments face numerous challenges, such as drug resistance [19] and impaired intestinal microecology [20], as depicted in Fig. 2. Despite HP's in vitro susceptibility to antibiotics, in vivo efficacy is frequently compromised, potentially due to the neutralization of antibiotics'bactericidal effects by gastric acid and mucus, as well as the formation of HP biofilms [19, 21, 22]. Standard eradication protocols, such as triple therapy and bismuth-containing quadruple therapy, demand the concomitant administration of several drugs over an extended period, typically 7–14 days [13, 14, 17, 18]. However, these conventional therapies are not without limitations. Firstly, they impose a significant burden on medication resources and economic costs. The latest national consensus report guidelines recommend an initial eradication regimen involving a 14-day continuous oral intake of a combination of"any bismuth compound + any PPI + amoxicillin + metronidazole", which significantly impacts public health and healthcare expenditures [11, 17]. Secondly, patient compliance is often inadequate, with treatment discontinuation due to a lack of understanding of the infection or side effects such as nausea, bloating, or discoloration of the stool or tongue coating [14, 18]. This not only increases the risk of treatment failure but also contributes to the development of antibiotic resistance in HP [14]. Furthermore, the issue of drug tolerance is becoming increasingly severe [19, 21, 22]. Long-term persistent infection may enable HP to form biofilms or adapt to antibiotic treatment through various mechanisms, such as modulating autophagy or forming intracellular bacteria, complicating the eradication of HP with antibiotics [19]. The most recent primary resistance rates for clarithromycin, metronidazole, levofloxacin, tetracycline, and amoxicillin in the Asia-Pacific region are 30%, 61%, 35%, 4%, and 6%, respectively [23]. In urban Chinese populations, resistance rates for clarithromycin and levofloxacin are particularly high, at 50.83% and 47.17%, respectively [24]. Lastly, the chronic administration of antibiotics can disrupt the gut microbiota, leading to dysbiosis within the intestinal ecosystem, implicated in a range of health issues, including immune dysfunction, metabolic disorders, and neurological pathologies [20, 25]. Even after treatment completion for more than 12 months, some patients still exhibit significant dysbiosis of the gut microbiota [20, 25].

The concept of Holistic Integrative Medicine (HIM) emphasizes a patient-centered approach that integrates multidisciplinary knowledge to address diseases through systemic observation and comprehensive evaluation of health outcomes [26]. In the context of HP management, HIM mandates therapies that not only eliminate the pathogen but also mitigate gastric mucosal injury, modulate inflammatory responses, and safeguard the gut microbiome—a delicate ecosystem pivotal to metabolic, immune, and neurological health [27]. While traditional antibiotic therapies disrupt this equilibrium, recent advances in biomaterials offer unprecedented opportunities to operationalize HIM principles [28]. Unlike broad-spectrum antibiotics, biomaterials enable spatiotemporally controlled antimicrobial action, mucosal repair, and microbiome protection through innovations such as pH-responsive drug release, biofilm-penetrating nanoparticles, and immune-modulating hydrogels [28–32]. For instance, pH-sensitive metal-organic frameworks selectively release bactericidal ions in the acidic gastric milieu while remaining inert in the neutral intestine, minimizing off-target effects [33]. Similarly, probiotic-loaded microspheres synergize pathogen suppression with microbiome restoration, exemplifying HIM’s dual therapeutic axis [34].

**Fig. 3:**
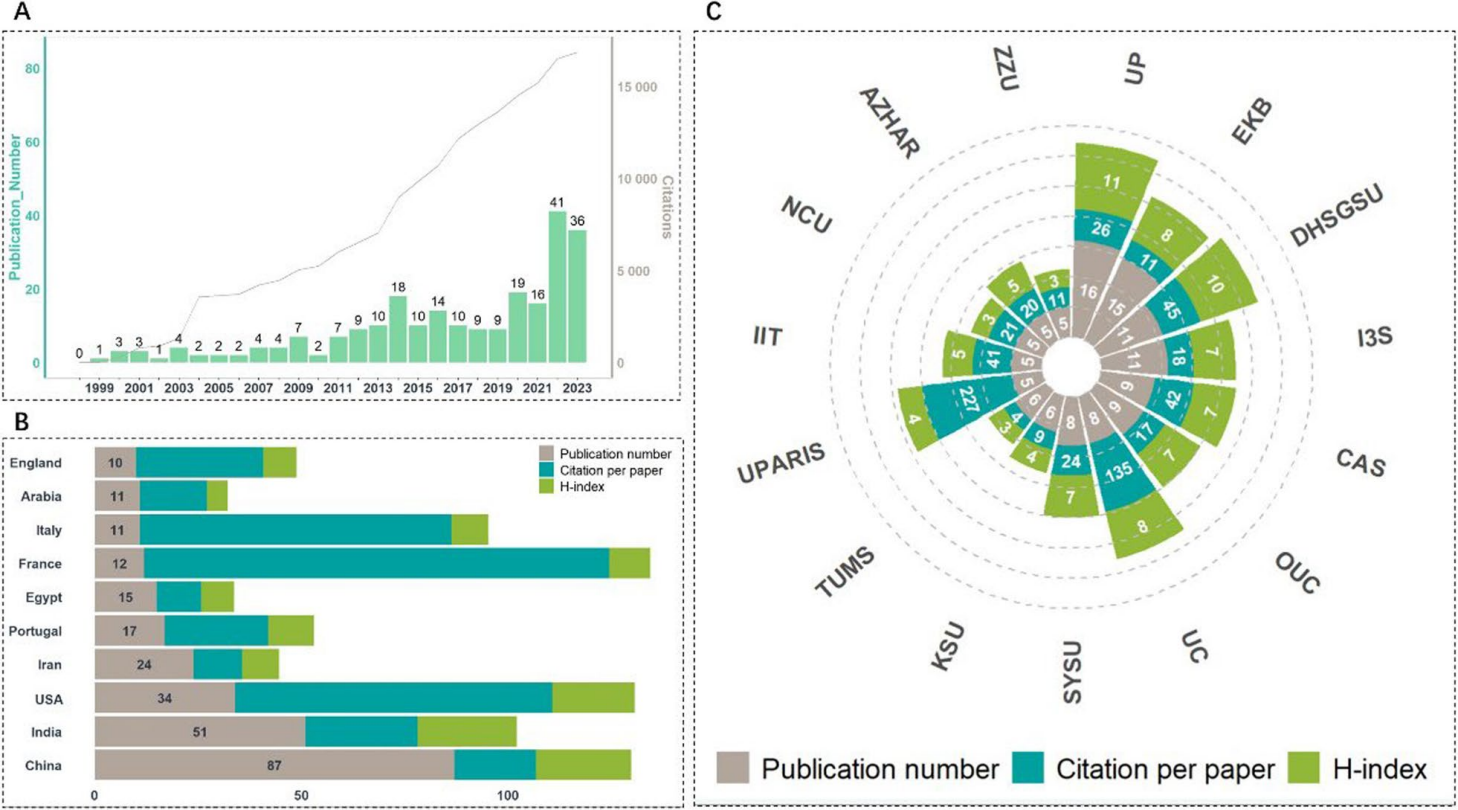
**A** The annual publication and citation growth of literature regarding biomaterials for HP treatment. **B** Most contributing countries/regions to the biomaterial-based HP treatment area. **C** The institutes contributing the most to the field of biomaterial-based HP treatment

**Fig. 4:**
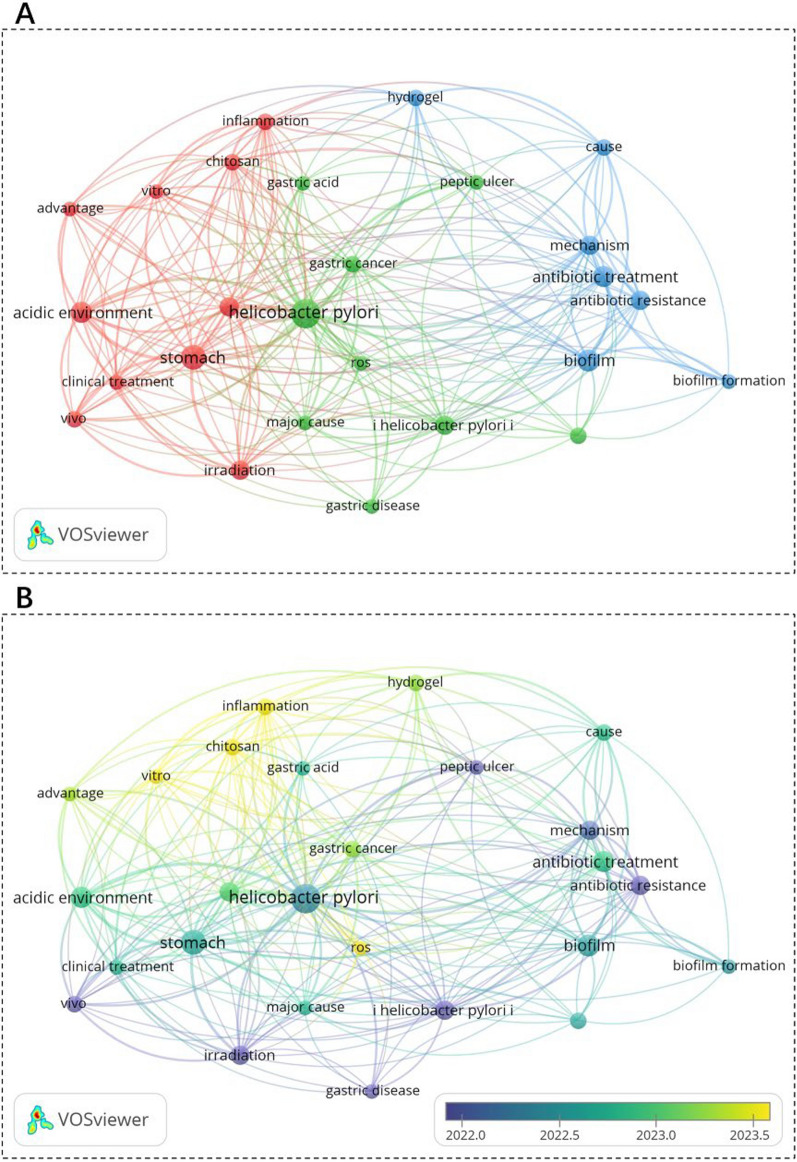
Co-occurrence analysis of author keywords with at least 8 occurrences. **A** Network visualization. **B** Overlay visualization with a timeline. The node size indicates the occurrence frequency; the node color represents the cluster; and the cluster resolution = 1.00

However, we analyzed the research trajectory of biomaterials in combating HP infection through bibliometrics (Figs. 3 and 4) and found that existing studies are still scattered and narrowly focused on material properties or single treatment methods. Few have systematically aligned these innovations with HIM’s integrative philosophy or addressed the translational challenges of balancing biocompatibility, targeting precision, and clinical scalability.

This review uniquely frames biomaterial innovation through the lens of HIM principles, proposing a paradigm shift from isolated pathogen eradication to multidimensional therapeutic integration. We hypothesize that engineered biomaterials, when synergized with multimodal therapies (*e.g.*, photothermal, sonodynamic), can achieve several goals aligned with the HIM framework [28, 32]. These include targeted HP elimination via mechanisms orthogonal to traditional antibiotics, such as reactive oxygen species (ROS) generation and ion interference. Additionally, these biomaterials can preserve the microbiome through spatial-temporal control of antimicrobial activity. Finally, they can modulate the host-microenvironment to attenuate inflammation and promote mucosal healing [28, 32]. Unlike prior studies focusing on material functionality alone, our approach systematically aligns biomaterial design with HIM’s core tenets-balancing efficacy, safety, and ecological harmony. For instance, stimuli-responsive hydrogels that release antimicrobial agents only at infected sites exemplify this dual focus on precision and biocompatibility, a synergy underexplored in earlier literature [34, 35].

**Scheme 1:**
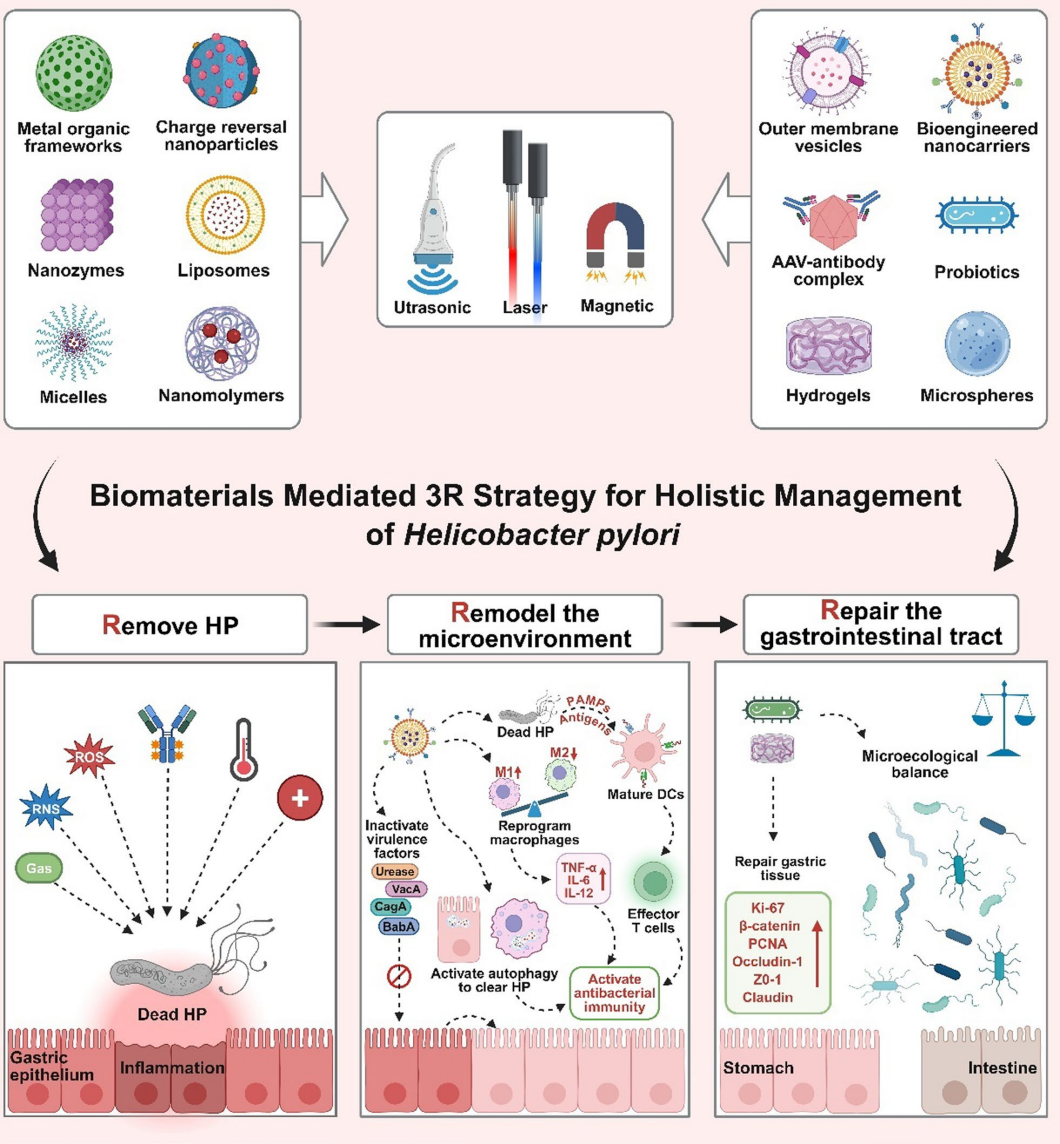
Biomaterials mediated 3R strategy for holistic management of HP. This approach utilizes various nanomaterials such as metal organic frameworks, charge reversal nanoparticles, and liposomes, activated by non-invasive stimuli like ultrasonic, laser, and magnetic fields. The"Remove"phase targets direct eradication of HP."Remodel"focuses on reshaping the immune microenvironment to clear pathogens."Repair"emphasizes gastric mucosa restoration and gut microbiota protection. This systematic intervention promises a patient-centered, sustainable treatment paradigm, enhancing therapeutic outcomes and combating antibiotic resistance. (Created with BioRender.com)

Building on this foundation, we introduce a novel integrative prevention and treatment paradigm for HP based on the 3R concept of nanomaterials (Remove, Remodel, and Repair). This paradigm aims to overcome the limitations of traditional therapies by directly combating antibiotic resistance to eliminate HP, reshaping the immune microenvironment to clear pathogens, and repairing gastric mucosa while protecting the gut microbiota. The nanomaterial-mediated 3R paradigm will drive HP management from a"simple killing"mode to a"systemic intervention"approach, achieving safe and effective integrated treatment of HP. By bridging the 3R concept with HIM philosophy and cutting-edge biomaterial engineering, this review charts a transformative path toward sustainable HP management, addressing gaps in patient-centered therapeutic design (Scheme 1).

## Clinical treatment dilemma of HP

In the early 1990 s, a milestone was reached with the establishment of a strategic approach to treating HP infections, termed"empiric therapy."This pioneering treatment protocol combined a proton pump inhibitor (PPI) with two antibiotics: clarithromycin and amoxicillin [17, 36]. The most recent national consensus, now in its fifth iteration, recommends a first-line eradication regimen for HP infections that comprises"any bismuth preparation + any PPI + amoxicillin + metronidazole", which is prescribed for a 14-day oral course [18, 37]. The Maastricht VI/Florence consensus report highlights standard bismuth quadruple therapy, which includes a PPI, bismuth, tetracycline, and metronidazole, and clarithromycin triple therapy, which consists of a PPI, clarithromycin, and amoxicillin, as frontline treatments for HP infections [10]. Although no treatment modality has yet achieved a 100% success rate, the consensus guidelines for HP eradication call for therapeutic options that can guarantee a minimum cure rate of 90% [10].

Despite these advancements, clinical treatment protocols are confronted with challenges such as patient noncompliance and worsening effectiveness due to increasing antibiotic resistance [23, 24]. Additionally, these treatments carry the risk of disrupting the delicate balance of the intestinal microbiome, potentially predisposing individuals to intestinal disorders [15, 20]. These interconnected issues collectively undermine treatment efficacy and exacerbate long-term health risks [28, 38, 39]. The pursuit of more effective and less invasive treatment strategies is therefore imperative to mitigate these challenges and improve patient outcomes in the management of HP infections.

### Poor compliance

Nonadherence to medication, frequently referred to as treatment noncompliance, is a critical issue in clinical practice and has a profound impact on treatment effectiveness [40]. The World Health Organization (WHO) focused on this matter in 2003, highlighting its enduring presence in long-term therapeutic plans [41]. With the aging of the global population, the severity of noncompliance has become increasingly significant [42, 43]. It is particularly associated with the use of multiple medications, known as polypharmacy, and greatly impedes the efficacy of treatments for a variety of conditions, including hypertension, asthma, and diabetes [44–46].

Compliance rates with HP eradication therapy are highly sensitive to the duration of treatment, underscoring the pivotal role of patient adherence in the success of HP eradication [47, 48]. Shahbazi et al. [49] reported a marked difference, with the success rate of eradication being 40 times greater in patients who demonstrated good compliance. The findings of Lan et al. [50], through logistic regression analysis, support this, showing that adherence to treatment is an independent predictor of successful eradication. Huguet et al. [40] further clarified the influence of compliance on eradication rates, noting that for first-line treatment, the rate is 89% for compliant patients compared with 58% for those who are not compliant. The rates of second-line treatment are 82% and 60%, respectively.

The standard prescription for HP infection typically involves a combination of at least three drugs, requiring strict adherence to the medication regimen over a 14-day period [51]. Concurrently, adverse reactions during the eradication process are significantly correlated with treatment failure and reduced compliance [52]. Some patients may independently decide to discontinue medication due to symptoms such as nausea and bloating during treatment or the discoloration of stool and tongue coating following the use of bismuth preparations [10]. It is estimated that approximately 10% of patients do not adhere to more than 60% of the prescribed dosage during the medication period [53]. These complexities pose considerable challenges for healthcare providers and patients as they navigate the therapeutic process.

### High resistance

The rapid increase in antibiotic resistance poses a significant threat, especially in the case of HP infections. These infections are characterized by high levels of resistance, including single-drug resistance (SDR), multidrug resistance (MDR), and heteroresistance (HR). The fundamental mechanisms underlying HP resistance to antibiotics can be broadly categorized into four types [19]: (1) Mutational alterations: Bacteria circumvent the effects of antibiotics by introducing mutations in their targets. (2) Reduced accumulation: The intracellular concentration of antibiotics is lowered by modifying efflux pumps or altering cell membrane permeability. (3) Enzymatic inactivation: Antibiotics are neutralized, or their activity is impaired through enzymes or virulence factors. (4) Escape mechanisms: Pathways that protect bacteria from being cleared by antibiotics are activated. Recent meta-analyses have underscored the high rates of primary resistance to antibiotics in the Asia–Pacific region, with rates of 30% for clarithromycin, 61% for metronidazole, 35% for levofloxacin, 4% for tetracycline, and 6% for amoxicillin [23]. In urban Chinese populations, resistance to clarithromycin and levofloxacin is particularly pronounced, with rates averaging 50.83% and 47.17%, respectively. [24] The presence of single-drug resistance in antibiotic-based triple therapy markedly diminishes the treatment success rate, with a 60% decrease in clarithromycin resistance, a 38% decrease in metronidazole resistance, and a 16% decrease in levofloxacin resistance [54, 55]. However, bismuth quadruple therapy combined with metronidazole has demonstrated a superior cure rate, even in patients with single-drug metronidazole resistance.

The increase in and persistence of SDR are largely due to pretreatment antimicrobial resistance (AMR) and the misuse and overuse of antimicrobial agents, which drive the ability of HPs to withstand antibiotic pressures [55]. MDR, which is characterized by resistance to three or more classes of antibiotics, is not well understood but is worsened by incorrect antibiotic prescriptions, poor patient compliance, and bacterial factors such as adaptive mutations, efflux pump upregulation, and biofilm formation [19]. The misuse and overuse of antibiotics, along with poor drug compliance, are significant factors contributing to MDR [56]. These elements create an environment that facilitates the survival and spread of drug-resistant HP strains [57].

Heteroresistance, where different bacterial populations within the same host respond differently to certain antibiotics, refers to the concurrent presence of resistant and susceptible strains of HP in the gastric mucosa. The existence of resistant strains heightens the risk of treatment failure and complicates therapy, even when susceptible strains are also present [58–60]. It is estimated that approximately 7–14% of HP infections exhibit HR, and various drugs, including levofloxacin, amoxicillin, and tetracycline, are linked to increased HR [58–60]. Current treatment guidelines for HP infection do not adequately address HR, and there is a lack of established treatment plans for infections featuring HR strains. AMR is the primary cause of treatment failure, presenting substantial challenges to clinical treatment strategies.

### Damage to the gut microbiota

The gut microbiota is instrumental in preserving intestinal equilibrium, and its disruption can have profound implications for human health [61]. The chronic administration of antibiotics to eradicate HP frequently leads to dysbiosis, an imbalance that may persist beyond 12 months posttreatment in some individuals [62, 63]. The consequences of such dysbiosis are far-reaching, impacting the immune, metabolic, and nervous systems and significantly jeopardizing overall health [64].

Current standard treatments for HP infections often employ a combination of antibiotics, PPIs, and either bismuth-containing or bismuth-free medications in triple or quadruple therapies. This practice has raised concerns about the potential to disrupt the gut microbiota [65]. Research suggests that approximately half of the patients who undergo these treatments experience a reduction in both the species count and the Shannon diversity index of their gut microbiota, resulting in a general decline in bacterial diversity following therapy [25].

The human gut microbiota is primarily composed of Firmicutes and Bacteroidetes [66]. Studies have shown that amoxicillin treatment can reduce Firmicutes levels in the gut, as observed in pregnant rats [67]. Oh et al. reported that standard 14-day triple therapy comprising lansoprazole, clarithromycin, and amoxicillin can decrease the relative abundance of Firmicutes while increasing that of Proteobacteria [68]. Similarly, quadruple therapy for HP eradication, which includes bismuth, can lead to dysbiosis, which is characterized by an increase in Proteobacteria and a decrease in Bacteroidetes and Actinobacteria [69].

The effects of short-term antibiotic treatments on the gut microbiota can be unexpectedly enduring. While microbiota diversity may eventually revert to pretreatment levels, these effects can linger for up to four years in some cases [70]. Posteradication therapy and gastrointestinal symptoms such as diarrhea and constipation are commonly reported [71]. There is considerable evidence that microbiota disruption is associated with conditions such as irritable bowel syndrome, inflammatory bowel disease, and *Clostridium difficile* infection [72]. PPIs can also modify the composition of the gut microbiota, with long-term use possibly resulting in small intestinal bacterial overgrowth (SIBO), *C. difficile*, and other intestinal infections [73].

The perturbation of the gut microbiota is a significant drawback of existing HP treatment protocols. There is an urgent call for innovative strategies for the treatment of HP infections that can effectively eliminate bacteria while also protecting the integrity of the gut microbiota.

### Limited by the immunosuppressive microenvironment

Antibiotic therapy faces significant challenges in combating HP infection, not only due to low eradication rates, high antibiotic resistance, and disruption of the gut microbiota, but also because of its inability to reverse the immunosuppressive microenvironment triggered by HP [74]. This microenvironment is characterized by the impairment of antigen presentation and phagocytosis functions, as described in a study integrating single-cell RNA sequencing and TCR profiling of cells from HP-positive individuals [74]. The infection leads to a monocyte-to-macrophage differentiation trajectory and broad functional impairment of T cell responses, potentially contributing to immune evasion by HP. The urgent need for novel therapeutic approaches is evident, with a focus on strategies that can reshape the immunosuppressive gastric ecosystem, thereby enhancing antibacterial immunity and treatment efficacy [28, 74].

Overall, the clinical treatment of HP faces significant challenges, including poor patient compliance, increasing antibiotic resistance, and disruption of the gut microbiota [19, 20, 40]. These issues are compounded by the immunosuppressive microenvironment triggered by HP, which impairs antigen presentation and phagocytosis, contributing to immune evasion [74]. Traditional antibiotic therapies often lead to low eradication rates, high resistance, and microbiome dysbiosis, further complicating treatment efficacy and long-term health outcomes [23–25]. Addressing these interconnected problems requires a holistic approach that integrates precision medicine, microbiome preservation, and patient-centered care [28].

## Bibliometric analysis of biomaterial treatment for HP

The persistent clinical challenges of antibiotic resistance, poor compliance, and microbiome disruption demand a paradigm shift toward innovative therapeutic strategies. To map the global research trajectory and identify emerging solutions, we conducted a comprehensive bibliometric analysis of biomaterial-based HP therapies, revealing how interdisciplinary innovations—from mucus-penetrating nanoparticles to microbiome-sparing multimodal platforms—are addressing these interconnected dilemmas.

### Data sources and methodological framework

We conducted a systematic bibliometric analysis using the Web of Science (WoS) core collection to map global research trends in biomaterial-based HP therapies from 1999 to 2024. The search strategy combined keywords related to biomaterials (*e.g.,* nanoparticles, hydrogels, microspheres) and HP infection (*e.g.,* Helicobacter pylori, Campylobacter pylori), yielding 279 relevant articles after refinement (196 research articles, 83 reviews) (Figure S1). Data were analyzed using VOSviewer (v1.6.17) for co-occurrence networks, Python (v3.10) for trend predictions, and R (v4.2.2) for statistical validation. Moreover, we further integrated machine learning (ML) models (Latent Dirichlet Allocation, LDA) to identify emerging research themes and validate keyword clustering patterns.

### Temporal and spatial trends in publications

We observed three distinct phases in annual publication growth (Fig. 3A): First is the Early Exploration phase (1999–2013), during which there was slow growth (1–5 articles/year), and it was dominated by foundational studies on metal nanoparticles (*e.g.*, bismuth compounds) and mucosal adhesion mechanisms. Next comes the Acceleration phase (2014–2020), in this phase, the annual output surged to 18–25 articles, mainly driven by advancements in stimuli-responsive nanomaterials (*e.g.*, pH-sensitive MOFs) and biofilm disruption strategies. The last phase is the Exponential Growth phase (2021–2024), which witnessed a record 41 publications in 2023, reflecting the intensified interest in multimodal therapies (*e.g.*, photothermal/immunomodulatory hybrids) and microbiome preservation.

Notably, we found that HP-related biomaterial research grew 2.3 × faster than the broader nanomedicine field (2014–2024), a conclusion validated by normalized publication counts against tumor-targeted nanotherapy data (*P* < 0.01, chi-square test). Geographically, China dominated with 87 articles (31.2% of total), followed by India (51) and the USA (34). Italy and France exhibited the highest citation impact (112.42 citations/paper), underscoring their influence in translational innovations (Fig. 3B, C and Table S1–3).

### Key research domains and AI-driven insights

Through co-occurrence analysis of author keywords, we identified three interconnected clusters (Fig. 4): Firstly, Gastric Barrier Engineering (Red Cluster), which is focused on overcoming mucus penetration challenges (*e.g.*, chitosan-modified nanoparticles) and acid stability (*e.g.*, ZIF-8@AP hydrogels). Then, Antibiotic Resistance Mitigation (Blue Cluster), which emphasizes non-antibiotic mechanisms (ROS generation, mechanical disruption) and resistance reversal via metal ions (Bi^3+^, Zn^2+^). Lastly, Complication Management (Green Cluster), which is aimed at inflammation modulation (*e.g.*, HSP70 upregulation) and microbiome preservation (*e.g.*, pH-dependent charge reversal nanomaterials).

Our ML-enhanced analysis also uncovered some understudied frontiers. Firstly, Emerging Theme 1 is about probiotic-biomaterial hybrids (*e.g.*, Lactobacillus-loaded liposomes) to competitively inhibit HP adhesion while restoring gut flora. Then, Emerging Theme 2 focuses on CRISPR-Cas9 functionalized nanoparticles for precise targeting of resistance genes (*e.g.*, 23S rRNA mutations). Lastly, the Prediction Model shows that logistic regression forecasts a cumulative 454 articles by 2030, with 60% focusing on combinatorial therapies (*e.g.*, SDT + autophagy activation).

### Limitations and future directions

While our bibliometric analysis highlights progress, we acknowledge critical gaps in three domains: clinical translation, material diversity, and standardization. A predominant challenge lies in bridging preclinical findings to clinical applications—only 5% of studies advanced beyond rodent models, largely hindered by unresolved nanotoxicity risks such as silver nanoparticle-induced neurotoxicity. Equally pressing is the field’s overreliance on metal-based systems (65% of publications), overshadowing innovative biomimetic platforms like extracellular vesicle mimics. Further complicating progress, inconsistent terminology (*e.g.*, interchangeable use of"nanoparticle"and"nanocarrier") introduces ambiguity in cross-study comparisons.

To address these gaps, we advocate prioritizing two strategic directions. HIM-aligned design should unify mucosal repair mechanisms (*e.g.*, EGF-loaded hydrogels) with microbiome modulation strategies (*e.g.*, prebiotic conjugates) within multifunctional platforms. Concurrently, AI-guided optimization—leveraging machine learning (ML) and deep learning (DL)—could refine nanoparticle-biofilm interaction predictions and dosing regimens. [75] Collectively, this AI-augmented framework not only delineates HP biomaterial advancements but also charts actionable solutions to overcome clinical translation barriers and accelerate therapeutic innovation.

## Biomaterials combat HP while protecting the gastrointestinal tract

The escalating antibiotic resistance and collateral damage to gut microbiota caused by conventional HP therapies necessitate the development of biomaterials that synergize antimicrobial efficacy with microbiome preservation. Guided by the principles of HIM, Advanced biomaterials—including nanoparticles, hydrogels, and microspheres—are engineered to overcome gastric barriers, target HP-specific virulence factors, and minimize ecological disruption in the gastrointestinal tract (Table 1). These biomaterials share commonalities and exhibit complementarities. Nanomedicines can augment the targeting of hydrogels and microspheres, while hydrogels and microspheres can increase the gastric dwell time of nanomedicines [28, 32, 76]. This section evaluates their design principles, mechanisms of action, and translational potential.

**Table 1:** Anti-HP mechanisms of biomaterials

Anti-HP mechanisms	Biomaterials	Examples	References
Resist stomach acid	Acid-resistant nanoparticles	Bi-MOF@CS-Se Cu-MOF@NF PtCo@G@CPB ICG@FCS	[77] [78] [79] [27]
	Acid-resistant hydrogels	Pd(H) @ ZIF-8 @ AP AP@CS@Lip@HKUST-1 C_12_G_2_ hydrogel *L.* reuteri@HTP	[80] [35] [81] [34]
	Acid-resistant microspheres	Leb-Mics	[82]
Penetrate the mucus layer	Liposomes	LipoLLA	[83]
	Polysaccharide	Cu-MOF@NF (FU) ICG@FCS (FU) AP@CS@Lip@HKUST-1 (CS) ZAN@CS MNDs (CS)	[78] [27] [35] [33]
Target HP	pH-Responsive nanozymes	MSPLNP-Au-CB FPB-Co–Ch	[84] [85]
	Dual-targeted cascade nanozymes	PtCo@G@H_2_A	[86]
	Protonated charge reversal nanoparticles	ZAN@CS MNDs	[33]
	Plasma membranes of gastric epithelial cells	AGS-NPs	[87]
	Polymyxin B-targeted polysaccharide (fucoidan)	ICG@FCS Cu-MOF@NF PtCu_3_-PDA@AIPH@Fucoidan	[27] [78] [88]
	Antibody-conjugated liposomes	GNS@Ab HpAb-Li-ICG	[89] [90]
	Genetically encoded crystal	Cry3 Aa-MIIA(D45E)-LL37-P17	[91]
Inhibition of urease activity	Metal ion generator	Bi-MOF@CS-Se (Bi^3+^) Cu-MOF@NF (Cu^2+^) ZAN@CS MNDs (Zn^2+^, Ag^+^) Pd(H)@ZIF-8@ AP (Zn^2+^) CS@Lip@HKUST-1 (Cu^2+^)	[77] [78] [33] [80] [35]
Destroy bacterial membranes and biofilms of HP	Surfactant liposomes	FU/ML-LA/EB NPs (EB) RLs@T780 TG (RLs) RHL@BP@ISL (RHL) CS@Lip@HKUST-1 (RHL) RHL/Cl-Ch-cal (RHL)	[92] [93] [94] [35] [95]
	Metal ion generator	Bi-MOF@CS-Se (Bi^3+^) Cu-MOF@NF (Cu^2+^) ZAN@CS MNDs (Zn^2+^, Ag^+^) Pd(H)@ZIF-8@ AP (Zn^2+^) CS@Lip@HKUST-1 (Cu^2+^)	[77] [78] [33] [80] [35]
	ROS generator	MSPLNP-Au-CB ZAN@CS MNDs FU/ML-LA/EB NPs RLs@T780 TG + Light ZnO_2_-Ce6@lipo + Light Ver-PLGA@Lecithin + US PtCu_3_-PDA@AIPH + US HpAb-Li-ICG + US Fe-HMME@DHA@MPN + US ICG@FCS + US	[84] [33] [92] [93] [96] [97] [88] [90] [98] [27]
	Heat generator	RHL@BP/ISL + Light GNS@Ab + Light RLs@T780 TG + Light FeCo@G@PEG + Magnetic FCSHMGNs + Magnetic	[94] [89] [93] [99] [100]
Against intracellular HP	Autophagy activator	FU/ML-LA/EB NPs (FU) AP@CS@Lip@HKUST-1 (PA) RHL/Cl-Ch-cal (cal) ICG@FCS + US	[92] [35] [95] [27]
Competitively inhibit HP	Probiotics	*L.* reuteri@HTP BLPs-SAM-FAdE (*L.* lactis)	[34] [101]
	Biomimetic membrane nanovesicles	OM-NPs OMVs/MMVs/hMVs	[102] [103]

### Nanoparticles

Nanoparticles possess a range of unique physicochemical properties, including precisely tunable minute sizes, high specific surface areas, and versatile functional architectures [104–107]. These attributes are pivotal in the development of innovative antimicrobial formulations designed to overcome the limitations typically associated with traditional antimicrobial therapies [28, 108, 109]. The antimicrobial effectiveness of nanoparticles is achieved through multiple mechanisms: (1) stimulus-triggered (pH, enzyme, et al.) release at targeted locations. (2) prevention of bacterial biofilm formation. (3) stimulation of the host’s innate and adaptive immune responses. (4) generation of ROS, heat, gas to combat HP.

#### Metal organic frameworks

Metal organic frameworks (MOFs) have emerged as a paradigm-shifting platform for HP therapy due to their structural versatility, high surface area, and pH-responsive drug release capabilities [77, 78]. These hybrid materials integrate metal nodes (*e.g.*, Zn^2+^, Cu^2+^, Fe^3+^) with organic linkers (*e.g.*, terephthalic acid, imidazolate) to create tunable pores that encapsulate antibiotics or metal ions [79, 80].

Zhou et al. developed an innovative bismuth-based MOF by leveraging bismuth ions as the metal nodes and trimesic acid as the organic ligand. Further functionalization with seleno-chitosan (CS-Se) yielded the nanodrug Bi-MOF@CS-Se [77]. This nanodrug employs charge interactions on the CS-Se outer layer to target mucins, facilitating mucosal adhesion and gastric retention. In vitro studies have shown that at pH = 2, Bi^3+^ is released more rapidly, increasing the protein leakage rate of HPs, which indicates the superior antibacterial activity of Bi-MOF@CS-Se under acidic conditions. In vivo experiments confirmed that Bi-MOF@CS-Se can effectively respond to gastric acid, with the exposed Bi-MOF releasing Bi^3+^ demonstrating significant antibacterial efficacy against both standard and clinically antibiotic-resistant HP strains. Importantly, the biosafety and stability of Bi-MOF@CS-Se in the intestine contribute to the preservation of gut microbiota integrity [77].

**Fig. 5:**
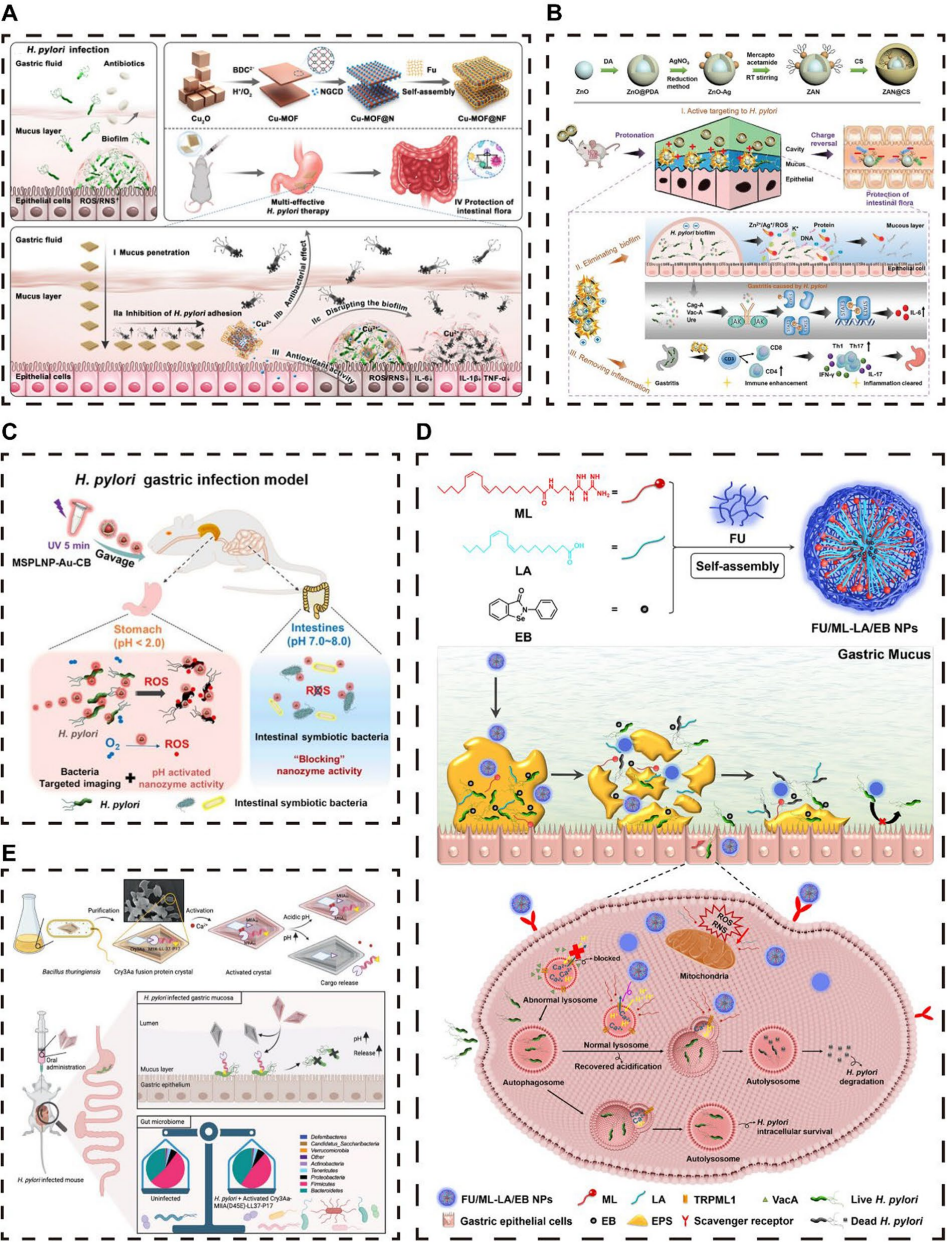
Nanoparticles resist HP and protect the intestinal microecology. **A** Metal‒organic frameworks (MOFs). **B** Protonated charge reversal nanodrugs. **C** pH-responsive persistent luminescence nanozyme. (D) Self-assembling Nanomicelles. (E) Genetically encoded antimicrobial crystal. **A **Reproduced with permission from ref, [78] © Shu C (2024). B Reproduced with permission from ref, [33]© Zou Y (2022). C Reproduced with permission from ref, [84] © Liu C (2023). D Reproduced with permission from ref, [92] © Yan L (2021). E Reproduced with permission from ref, [91] © Zhang W (2023)

A mucin-penetrating therapeutic platform, Cu-MOF@NF, consisting of a copper-based MOF loaded with nitrogen-doped carbon dots (NGCDs) and the naturally active polysaccharide fucoidan (FU), was introduced (Fig. 5A) [78]. This platform can penetrate the mucus layer and prevent HPs from adhering to gastric epithelial cells. The Cu^2+^ ions released by Cu-MOF can degrade polysaccharides within the biofilm, disrupting the"planktonic to bacterial biofilm"cyclic growth pattern and thus preventing recurrent and persistent infections. Cu-MOF@NF has shown the ability to eliminate biofilms and combat HP, including multidrug-resistant strains, while maintaining the balance of the gut microbiota [78].

#### Charge reversal nanoparticles

Charge reversal nanoparticles exploit the acidic gastric environment to enhance bacterial targeting [86]. Liu et al. successfully developed an innovative protonated charge-reversal metal-based nanodrug (MND) known as ZAN@CS MND (Fig. 5B) [33]. These ZnO-Ag nanoparticles, which are functionalized with thioglycolic acid, have acquired the ability to undergo reversible charge transition, resulting in ZAN, a potent antibacterial agent. This functionalization endows ZAN with the unique feature of charge reversal. The use of chitosan to modify ZAN enhances its mucosal targeting efficacy and ability to penetrate the mucosal barrier, amplifying its bactericidal impact. In the acidic environment of the stomach, ZAN becomes protonated and positively charged, actively targeting negatively charged bacterial membranes to eradicate bacteria and biofilms through the release of metal ions (Zn^2+^ and Ag^+^) and reactive oxygen. In contrast, at the neutral pH of the intestinal environment, ZAN has a negative charge, which minimizes interactions with the gut microbiota, preserving its abundance, functionality, and species diversity [33].

#### Nanozymes

Since the serendipitous discovery in 2007 that iron oxide nanoparticles exhibit peroxidase-like activity, a plethora of nanomaterials have been identified to possess enzyme-mimetic activities [110–112]. These nanomaterials, termed nanozymes, offer a spectrum of advantages over their natural counterparts, including cost-effectiveness, enhanced stability, ease of large-scale production, and multifunctionality [84–86]. Notably, under acidic conditions, certain nanozymes have demonstrated superior peroxidase and oxidase activities compared with natural enzymes, preabsorbing protons and catalyzing the decomposition of hydrogen peroxide (H_2_O_2_), thereby generating highly reactive antimicrobial substances such as reactive oxygen species (ROS), which are crucial for bacterial eradication [84–86]. Owing to these attributes, nanozymes have emerged as a novel class of antimicrobial agents that are extensively utilized in a variety of antimicrobial applications [28, 84–86]. Despite their promise, the short half-life of ROS (approximately 3.5 microseconds) and their limited diffusion range (hundreds of nanometers) can hinder their interaction with bacteria, highlighting the necessity for surface modifications to increase their antimicrobial efficacy. The targeting of specific bacterial receptors is achieved through common surface modification techniques, such as the conjugation of nanozymes with bacterial molecules (*e.g.*, bacterial adapters, phenylboronic acid, polymers, and biomacromolecules) and electrostatic cross-linking [95]. These modifications enable the specific capture of HPs by biomaterials, increase the local concentration of ROS around bacteria to increase their anti-HP potency, and confer biomaterials with the ability to target HPs, thereby facilitating the preservation of the symbiotic flora of the gastrointestinal tract.

Phenylboronic acid, a molecule capable of reversibly binding to peptidoglycan on bacterial cell walls, has been employed for the specific capture of target bacteria [84]. Yan et al. [84] synthesized a chitosan-phenylboronic acid (CB) conjugate, linking the amine groups of chitosan with the carboxyl groups of 4-carboxyphenylboronic acid, which achieved targeted adhesion to HP surfaces. They further developed a multifunctional nanozyme, MSPLNP-Au-CB, composed of mesoporous silica-coated persistent luminescent nanoparticles (MSPLNPs), gold nanoparticles (AuNPs), and chitosan-phenylboronic acid (CB) (Fig. 5C) [84]. This nanozyme, under acidic conditions in the stomach, targets HP, activates its enzymatic activity, and catalyzes the formation of ROS, effectively inactivating HP due to the high local concentration of ROS. Importantly, nanozyme activity is inhibited in the neutral environment of the intestine, preventing nonspecific damage to gut commensal bacteria. Additionally, the material leverages the long-lasting luminescence of MSPLNPs for imaging purposes, eliminating the need for exogenous fluorescent labeling [84]. Zhang et al. [79] developed a novel pH-responsive graphite nanoenzyme, PtCo@G@CPB, which consists of PtCo nanocrystals and bacterial anchoring molecules and is specifically designed for the selective treatment of HP. Under acidic conditions, PtCo@G@CPB exhibited peroxidase-like activity, generating superoxide radicals (O_2_^•−^) that attack the bacterial membrane of HPs, leading to cytoplasmic leakage and bacterial death. Crucially, the activity of these nanozymes is attenuated at neutral intestinal pH, thereby minimizing side effects on healthy tissues and commensal bacteria [79].

#### Liposomes and micelles

Liposomes are phospholipid-bilayer nanovesicles enabling targeted drug delivery, widely explored for enhancing antimicrobial efficacy against HP infections [35, 83]. Lai et al. [35] developed a multifunctional liposomal platform, CS@Lip@HKUST-1, for antibiotic-free eradication of HP. The system integrates a copper-based MOFs (HKUST-1) encapsulated in a lipid layer containing rhamnolipid (RHL), phosphatidic acid (PA), and cholesterol, further coated with chitosan and embedded in an ascorbyl palmitate hydrogel. RHL disrupts HP biofilms by binding extracellular matrix metal ions and inhibiting biofilm-associated signaling. PA promotes lysosomal Ca^2+^ efflux, restoring host autophagy to eliminate intracellular bacteria. The positively charged CS enhances mucosal adhesion and gastric retention, while pH-responsive Cu^2+^ release from HKUST-1 disrupts bacterial membranes and inhibits urease activity. In vivo studies demonstrated significant reduction of HP burden without disturbing gut microbiota diversity, highlighting its potential as a targeted, microbiota-sparing therapeutic strategy against resistant infections [35].

Nanomicelles are amphiphilic polymer-based nanoparticles with hydrophobic cores, optimizing drug solubility and site-specific delivery for combating HP infections [92, 94]. Zou et al. developed fucoidan-coated self-assembled nanomicelles (FU/ML-LA/EB NPs) that encapsulate the urease inhibitor ebselen, providing an antibiotic-free strategy to combat HP (Fig. 5D) [92]. These nanomicelles penetrate the gastric mucus, disrupt biofilm structures, and enter bacterial cells to inhibit urease activity, effectively killing both extracellular and intracellular HP. Additionally, they mitigate oxidative stress, potentially reducing gastric damage and carcinogenesis pathways. In vivo studies have shown that this treatment significantly reduces the HP burden, suggesting a promising therapeutic approach for HP infections [92].

#### Outer membrane vesicles

Outer membrane vesicles (OMVs), naturally secreted by Gram-negative bacteria, are emerging as biocompatible drug carriers [102, 103, 113]. A landmark study by Gu et al. [102] engineered OMVs derived from *E.* coli to encapsulate clarithromycin. The OMVs selectively fuse with HP membranes via lipid bilayer interactions, delivering antibiotics intracellularly. This approach reduced the clarithromycin dose required for eradication by tenfold in vitro and showed negligible impact on gut microbiota in vivo [102].

Additionally, Fan et al. [114] developed CLR-Ag^+^@MON@HA@OMV (CAMO), an acid-responsive nanocarrier. OMV significantly enhances the bio-safety and therapeutic efficacy of CAMO. Experiments show that OMV protects gastric mucosal cells, boosting their viability to 100% versus 85% for CAM. In mice, CAMO normalizes levels of IL-6, TNF-α, and IL-1β, showing stronger anti-inflammatory effects than triple therapy and CAM. Moreover, CAMO-treated mice maintain gut microbiota diversity, unlike those under triple therapy. Thus, OMV plays a crucial role in CAMO's safe and effective treatment of HP infections [114].

Liu et al. [103] developed yogurt-inspired hybrid membrane vesicles (hMVs) to combat HP infection. The hMVs, combining bacterial outer membrane vesicles and modified milk fat globule membrane vesicles, effectively reduced HP adhesion and promoted gastric mucosal repair. Notably, hMVs enhanced macrophage phagocytosis of apoptotic epithelial cells, significantly increasing the binding rate and phagocytosis ratio of macrophages to apoptotic cells. This dual mechanism not only inhibited bacterial colonization but also maintained gastric mucosal integrity, offering a promising antibiotic-free therapeutic strategy [103].

#### Bioengineered nanocarriers

The integration of synthetic biology and nanomaterial engineering has led to innovative antimicrobial platforms [86, 91, 115, 116]. Deng et al. [86] developed PtCo@G@H_2_A, a dual-targeting nanozyme that enhances targeting of HP by 850% compared to single-targeting strategies. This nanozyme combines hematin and L-arginine to bind HP's receptor protein and capture it via protonation in the acidic gastric environment. It catalyzes the generation of ROS and nitric oxide, effectively treating HP while neutralizing its surface charge in the intestinal fluid to protect gut microbiota [86].

Zahra Ahmadzadeh Chaleshtori et al. [115] constructed a recombinant plasmid containing the HP LeoA gene and conjugated it with chitosan nanoparticles to immunize BALB/c mice. Using the Vaxign tool to analyze HP genomes, they selected the outer membrane as a vaccine candidate. The nanovaccine induced higher levels of LeoA-specific IgG and TNF-α in mice, achieving 87.5% immune protection and reducing gastric damage and HP load. Activated CD3^+^ T cells inhibited gastric cancer cell growth, demonstrating the vaccine's potential immunotherapeutic benefits [115].

Zhang et al. [91] engineered a genetically encoded antimicrobial crystal, Cry3 Aa-MIIA-AMP-P17, which selectively targets HP (Fig. 5E). The crystal protects antimicrobial peptides (AMPs) from gastric degradation, releasing them in the stomach to disrupt HP membranes. In murine models, it achieved > 99% bacterial reduction while maintaining 95% gut microbiota diversity, outperforming conventional therapies. Its pH-responsive release and receptor-specific binding minimize off-target effects, making it a promising microbiome-sparing alternative for HP eradication [91].

#### Challenges and future directions of anti-HP nanoparticles

The surge in drug-resistant bacteria is alarming, concurrently eroding the availability of effective treatment options. This scenario highlights the urgent need for innovation in the development of novel antimicrobial agents. Extensive research has underscored the potential of nanoparticles as carriers for antimicrobial drugs, significantly enhancing the bioavailability and therapeutic efficacy against HP for frontline antibiotics such as amoxicillin, clarithromycin, and metronidazole [105–107]. Despite these achievements, the current approach has not yet addressed the risk of disrupting the gut microbiota. The advent of nanoparticles that exhibit antimicrobial activity in acidic environments, along with their tailored enhancement of anti-HP capabilities while avoiding adverse effects on the gut flora, represents a promising therapeutic strategy for HP infections.

However, the extensive specific surface area and pronounced chemical reactivity of nanoparticles are not without risk; they are capable of generating ROS that can penetrate cells and tissues, posing the threat of irreversible oxidative stress and organelle damage [117]. Notably, numerous in vitro and in vivo studies have demonstrated that certain metal nanoparticles, such as silver nanoparticles (AgNPs), have a heightened likelihood of crossing the blood‒brain barrier, entering the brain, interacting with neuronal components, and causing neurotoxic effects [118].

Therefore, the clinical application of nanoparticles in the treatment of HP requires a thorough and systematic investigation, encompassing a detailed assessment of their potential adverse effects and nanotoxic potential. It is essential to design a new generation of antimicrobial formulations that are not only effective and safe for clinical use but also protective of the gut microbiota, thereby preventing the onset of intestinal disorders related to dysbiosis.

### Hydrogels

**Fig. 6:**
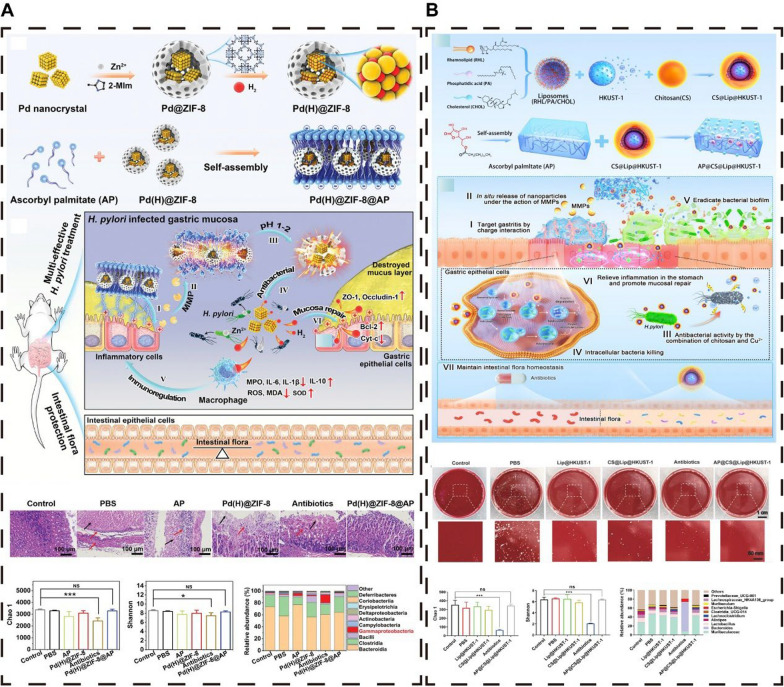
Hydrogels offer a promising defense against HP while maintaining the integrity of the intestinal microbiota. **A** Hydrogels serve as a protective barrier for metal‒organic frameworks (MOFs), shielding them from the corrosive effects of gastric acid, thereby enabling their antimicrobial action against HP and preserving the balance of the intestinal microecology. **B** By facilitating targeted delivery and localized release of nanoparticles, hydrogels mediate autophagy regulation, which is instrumental in the complete eradication of HP and the safeguarding of the intestinal microbiota. **A **Reproduced with permission from ref, [80] © Zhang W (2022). **B** Reproduced with permission from ref, [35] © Lai Y (2024)

Hydrogels, three-dimensional polymeric networks with hydrophilic properties, are crafted through chemical or physical cross-linking of water-soluble polymers [119–121]. Research indicates that HP infection incites chronic inflammation in the gastric mucosa, characterized by the upregulation of positively charged proteins and matrix metalloproteinases (MMPs), creating a site of inflammation with a positive charge [35]. Ascorbyl palmitate (AP), a compound that is stable under acidic conditions due to its hydrophilic ascorbic acid and lipophilic palmitic acid groups, forms negatively charged hydrogels. These AP hydrogels can specifically target inflammatory sites and facilitate drug release via MMP-mediated hydrolysis, offering an innovative therapeutic strategy for HP infection through the release of antimicrobial agents [35]. For example, Zhang et al*.* developed a novel biomaterial that integrates a metal‒organic framework, Pd(H)@ZIF-8, with a negatively charged AP hydrogel (Fig. 6A) [80]. This formulation encapsulates hydrogen-producing Pd(H)@ZIF-8 within the AP hydrogel, enabling targeted delivery and on-demand release at sites of gastric inflammation. Upon decomposition by gastric acid, Pd(H)@ZIF-8 generates Zn^2+^ ions and releases hydrogen gas, which alters cell membrane permeability and inhibits urease activity, leading to the eradication of HP. This approach results in significantly fewer side effects on the gut microbiota than traditional antibiotic therapies do, effectively preventing dysbiosis [80].

Similarly, we reported a nonantibiotic hydrogel material based on a multifunctional organic copper framework (HKUST-1): AP@CS@Lip@HKUST-1 (Fig. 6B) [35]. This material, which is composed of a lipid layer containing phosphatidic acid (PA), rhamphoyl lipid (RHL), and cholesterol (CHOL) encapsulated in chitosan (CS), is then loaded into an AP hydrogel. The innovative material aggregates at the gastric inflammatory site through electrostatic attraction, and the MMP degrades the hydrogel to release CS-encapsulated nanoparticles that target and kill HPs through chitosan-mediated action and copper ion release. Additionally, RHL disrupts biofilms through its surfactant action, and PA promotes lysosomal acidification and activates host autophagy to clear intracellular HP. Moreover, AP@CS@Lip@HKUST-1 alleviated inflammation and enhanced mucosal repair by delaying Cu^2+^ release while protecting the gut microbiota [35].

As an effective delivery system for HP treatment agents, hydrogels exhibit excellent biocompatibility, degradability, and controllable drug release capabilities [81]. Antimicrobial peptides (AMPs) offer a unique advantage in combating drug-resistant bacteria because of their rapid disruption of bacterial cell membranes and their anti-inflammatory and immunomodulatory properties. Gong et al*.* selected the amphiphilic C12G2 antimicrobial peptide and prepared a C12G2 hydrogel, C12-G(IIKK)2I-NH2(C12G2), based on PBS for the oral treatment of HP infection [81]. C12G2 molecules can be released under acidic stomach conditions, forming an alpha-helical structure that rapidly kills HPs through a membrane disruption mechanism, modulating the immune response by downregulating proinflammatory cytokines and upregulating anti-inflammatory cytokines, thereby increasing therapeutic efficacy. Under acidic conditions (pH = 4), the C12G2 hydrogel completely prevented HP infection within three days. Compared with the unassembled C12G2 hydrogel, the C12G2 hydrogel demonstrated superior biocompatibility and protease stability. Compared with antibiotic treatments, the C12G2 hydrogel not only kills bacteria rapidly but also has unique immunomodulatory functions. AMP hydrogels can swiftly eliminate pathogens in gastric fluid and do not require the use of antibiotics or PPIs during treatment. This work paves the way for the future development of safer, more convenient, and more effective alternatives to antibiotics [81].

Hydrogels have become a cornerstone in the field of antimicrobial medical materials, largely owing to their unique three-dimensional network structure, exceptional hydrophilicity, and outstanding biocompatibility. When infused with antibiotics, these hydrogels can significantly diminish the dependency on conventional antibiotic treatments [122]. Compared with the oral antibiotics used in isolation, these antibiotics have also attracted considerable attention in the realm of HP eradication, demonstrating superior antimicrobial potency [123, 124]. However, concerns regarding the potential adverse effects on the gut microbiota cannot be completely overlooked. The emergence of hydrogels that incorporate novel antimicrobial agents free from antibiotics represents a significant step forward in addressing challenges such as antibiotic resistance. Notably, antimicrobial peptide (AP) hydrogels are capable of selective hydrolysis at infection sites with elevated matrix metalloproteinase (MMP) activity, enabling the targeted delivery of antimicrobial agents to combat HP while preserving the balance of the gut microbiota. Nonetheless, the cytotoxicity and environmental risks associated with chemical crosslinkers commonly used in hydrogel synthesis are critical issues [125]. Therefore, there is an urgent need to innovate and develop environmentally friendly, nontoxic crosslinking agents and appropriate delivery systems. As research advances, we hope that innovative solutions will be devised to overcome these obstacles. This will lead to the creation of antimicrobial hydrogels that are more environmentally friendly, safer, and more effective [126–128].

### Microspheres

Microspheres, as uniform monolithic entities with diameters ranging from 0.1 to 1000 μm, have become a cornerstone in the pharmaceutical industry for the development of controlled drug release systems [129]. They provide exceptional drug loading capacities and the ability to modulate drug release kinetics, which can markedly decrease the required dosage while maintaining therapeutic efficacy over an extended duration [130]. Chitosan, a naturally occurring biocompatible polysaccharide, is distinguished by its -NH_2_ groups, which become protonated under acidic conditions. This protonation enables electrostatic interactions with the negatively charged gastric mucin and bacterial membranes, thereby endowing chitosan with mucoadhesive properties [131, 132]. Moreover, chitosan has antimicrobial activity against a broad spectrum of bacteria, including HP [133–135]. Gonçalves et al. [82] ingeniously engineered chitosan microspheres decorated with Lewis b glycans (Leb-Mics), which specifically target HP strains expressing BabA/SabA adhesins. This targeted interaction hinders or alleviates the adhesion of HP to the gastric mucosa, which contains the corresponding glycans [82]. These chitosan microspheres, which are two sizes (XL, approximately 120 μm, and XS, approximately 40 μm) and have varying degrees of acetylation (6% and 16%), adhere to HP strains without inducing cytotoxicity to gastric cells. In a murine model of HP infection, treatment with XL6 and XS6 chitosan microspheres resulted in an 88% reduction in infection, with the microspheres being safely cleared through the gastrointestinal tract following oral administration [136]. This strategy represents a significant step toward an antibiotic-free approach for the management of HP infections.

The high drug-loading capacity of microspheres, in conjunction with their sustained release profile, augments the efficiency of drug delivery against HP, ensuring that bactericidal concentrations are sustained. The possibility of integrating microspheres with other materials to construct a multifunctional anti-HP platform presents a promising and innovative alternative for the eradication of HP infections.

### Probiotics

Probiotics are defined as live microorganisms that confer a health benefit on the host when administered in adequate amounts [137, 138]. In the context of HP eradication, probiotics have garnered significant attention [139, 140]. Traditional treatments for HP primarily rely on antibiotics, which are increasingly challenged by antibiotic resistance and disruption of gut microbiota homeostasis [28, 141]. Consequently, alternative therapies are urgently needed. Probiotics, such as Lactobacillus reuteri, can inhibit HP growth and alleviate gastric inflammation by competing for adhesion sites, secreting antimicrobial substances, and modulating host immune responses, thus offering a promising alternative for HP management [34, 101].

A comprehensive analysis of 36,699 treatments reveals that probiotics prescribed with HP eradication therapy in Europe enhance effectiveness (OR 1.631 [95% CI 1.456–1.828]) and safety, with lower severe adverse events (1.06% and 1.90%) [139]. Lactobacillus boosts treatment efficacy, while Bifidobacterium and Saccharomyces improve safety profiles [139].

Lai et al. [34] present a novel hydrogel-transformable probiotic powder (*L.* reuteri@HTP) based on *Lactobacillus reuteri* for targeted eradication of HP. The powder, composed of *L. reuteri*, hyaluronic acid (HA), tannic acid (TA), and polyvinyl alcohol (PVA), rapidly transforms into a hydrogel upon contact with water. This transformation enhances the survival of *L. reuteri* in the harsh gastric environment and ensures selective release at HP-infected inflammatory sites. *L. reuteri* targets and reduces HP colonization while secreting reuterin to eliminate the bacteria. Additionally, TA’s antioxidant properties help alleviate inflammation, and HA supports gastric mucosal repair. The *L.* reuteri@HTP powder remains stable at room temperature for at least six months and does not disrupt the gut microbiota, making it a promising alternative to traditional antibiotics for HP treatment [34].

A mucosal vaccine derived from probiotics, BLPs-SAM-FAdE, has been developed by Zhang et al. to combat HP [101]. This vaccine leverages bacteria-like particles (BLPs) from *Lactic* acid bacteria (*L.* lactis) to display a multi-epitope antigen SAM-FAdE targeting M cells with 90% efficiency. Upon oral immunization, BLPs-SAM-FAdE effectively targets M cells in mouse Peyer's patches (PPs), facilitating the transport of particulate vaccines to dendritic cells (BMDCs) and stimulating their maturation. This process significantly increases the proportion of plasma cells and germinal center B cells, indicating robust mucosal and humoral immune responses. Notably, BLPs-SAM-FAdE induces notable antigen-specific sIgA production, CD4^+^ T cell responses (Th1/Th2/Th17), and serum IgG production. The vaccine also dramatically reduces HP adhesion and specific 16S rRNA expression in gastric mucosal tissue, protecting gastric tissue from damage [101].

Overall, this chapter underscores the potential of biomaterials to combat HP while safeguarding the gastrointestinal tract. Engineered nanoparticles, hydrogels, and microspheres leverage pH-responsive drug release, ROS generation, and biofilm disruption to precisely eliminate HP, aligning with the 3R paradigm. The"Remove"phase targets direct eradication of HP, while"Remodel"reshapes the immune microenvironment by modulating macrophages, DCs and T cells to clear pathogens."Repair"emphasizes gastric mucosa restoration and gut microbiota protection. These innovations integrate pathogen eradication with mucosal repair and microbiota protection, implementing the principles of HIM by balancing efficacy, biocompatibility, and ecological harmony. Despite progress, challenges remain in clinical translation and material diversity. Future directions include multimodal therapies integrating photothermal, sonodynamic, and magnetic strategies to enhance spatiotemporal control.

## Multimodal therapy against HP to safeguard the gastrointestinal tract

Biomaterials that exhibit sensitivity to external stimuli, including light, ultrasound, and magnetic fields, have demonstrated remarkable antimicrobial potential. Phototherapeutic agents, for example, upon laser irradiation, are capable of producing ROS or inducing thermal effects, both of which are efficacious in neutralizing microbial pathogens [28, 142–144]. By judiciously applying these conditions to the gastric milieu, biomaterials can be engineered to deploy antimicrobial substances that precisely target and eradicate HP while remaining inert in the unstimulated intestinal tract, thus safeguarding the gut microbiota [28, 145]. The activation and sustenance of the antimicrobial response can be meticulously regulated by modulating the intensity and duration of the stimuli, thereby increasing the temporal and spatial specificity and augmenting therapeutic efficacy while curtailing unintended side effects [32, 146]. The convergence of biomaterials with cutting-edge technologies such as laser therapy, ultrasound, and magnetic fields represents a pioneering approach in the battle against HP infections. This strategy is dual-pronged, aiming for the extermination of HPs while vigilantly preserving the intestinal microflora, which is integral to the maintenance of gastrointestinal health (Table 2). The evolution and deployment of these biomaterials necessitate an intricate comprehension of their dynamic interactions within the biological milieu and the fine-tuning of their antimicrobial attributes. The prospect of integrating stimuli-responsive biomaterials into the antimicrobial therapeutic landscape is expansive and promising for developing more efficacious and precise treatments for HP infections.

**Table 2:** Summary of multimodal therapies

Material	Synergistic mode	Action mechanism	References
RHL@BP/ISL	PTT	Multifunctional RHL@BP/ISL nanodrug synergizes with PTT for efficient eradication of drug-resistant HP, overcoming acidity and biofilm barriers	[94]
GNS@Ab	PTT, PA	GNS conjugated with pH-sensitive antibodies target *H. pylori*, enabling PA imaging and PTT for eradication without disrupting gut microbiota	[89]
ZnO_2_-Ce6 @lipo	PDT, CDT	ZnO_2_-Ce6@lipo nanocomposite generates ROS via PDT and CDT, neutralizes stomach acid, and eradicates HP with minimal impact on gut microbiota	[96]
RLs@T780 TG	PTT, PDT	RLs@T780 TG nanomedicine penetrates gastric mucus, targets MDR HP, and combines PTT and PDT for effective eradication without disrupting gut flora	[93]
Ver-PLGA @Lecithin	SDT	Ver-PLGA@Lecithin nanoparticles neutralize HP toxins and, with ultrasound, generate ROS for sonodynamic inactivation, preserving gut microbiota balance	[97]
PtCu_3_-PDA @AIPH @Fucoidan	SDT	The PPAF sonodynamic nanocomposite penetrates mucus, targets HP, disrupts biofilms, and eradicates bacteria via ROS and R• under ultrasound, preserving microbiota balance	[88]
HpAb-LiP-ICG	SDT, PA	HpAb-LiP-ICG liposomes target HP for PA imaging and SDT, utilizing ultrasound to activate ICG, generating ROS for eradication with minimal tissue toxicity	[90]
Fe-HMME@ DHA@MPN	SDT, CDT	Fe-HMME@DHA@MPN nanogenerators self-enhance ROS production via SDT and CDT, eradicating drug-resistant HP and biofilms with minimal impact on gut microbiota	[98]
FeCo@G@PEG	MHT	FeCo@G@PEG nanoheaters, under alternating magnetic fields, upregulate HSP70 to combat HP, with efficient magnetothermal therapy and rapid excretion	[99]
FCSHMGNs	MT, CDT	Single-iron microsweepers navigate dynamically, adhering to walls, increasing interactions with HP, and inhibiting it via acid-triggered ROS generation	[100]

### Phototherapy

**Fig. 7:**
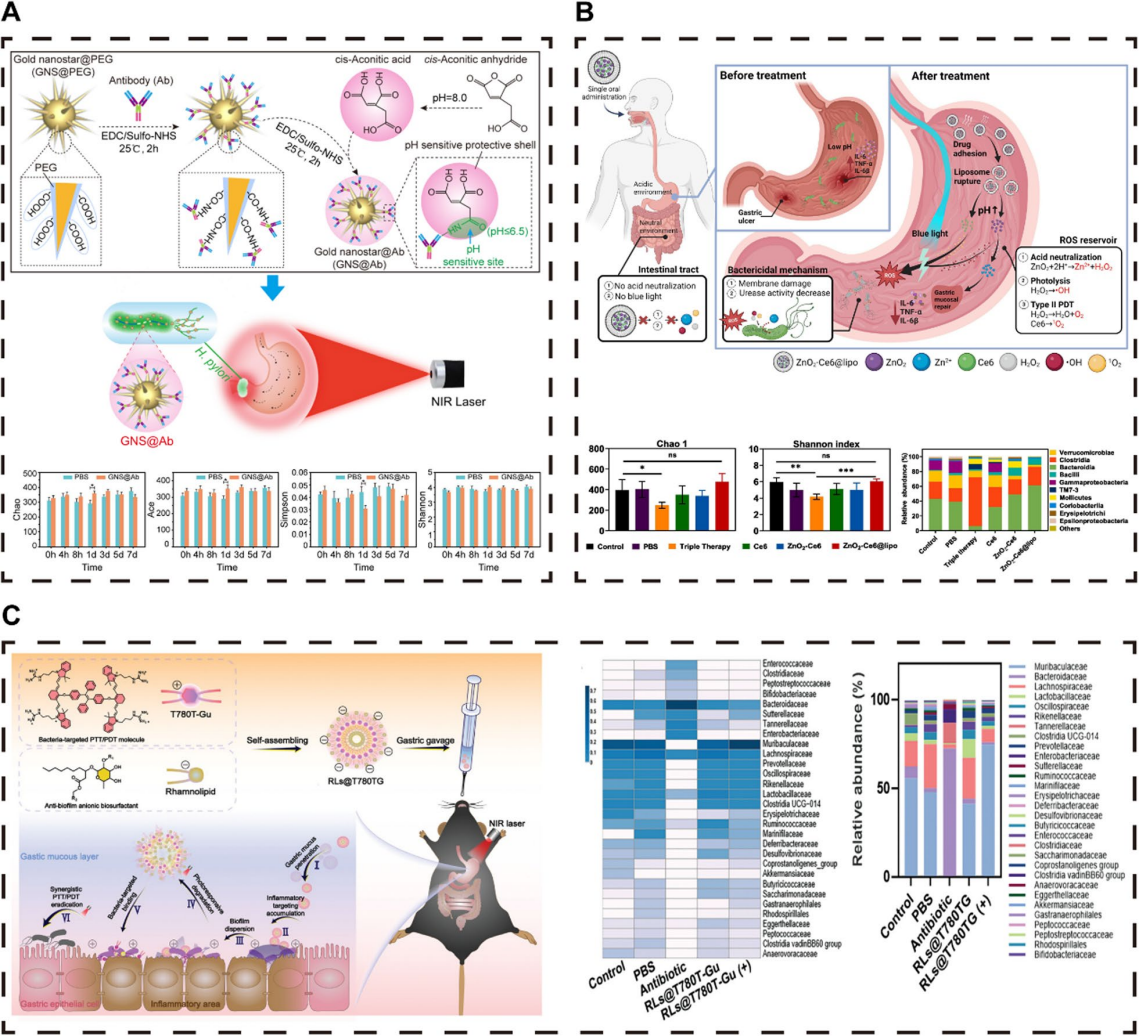
Biomaterial-mediated PTs resist the HP and protect the intestinal microecology. **A** Multifunctional liposome nanoparticle-mediated PTT. **B** Metal-based nanozyme-mediated PDT. **C** Multifunctional PSs that jointly mediate PTT and PDT. **A **Reproduced with permission from ref, [89] © Wang R (2022). B Reproduced with permission from ref, [96] © Wong K (2024). C Reproduced with permission from ref, [93] © Qiao Y (2024)

In the vanguard of antimicrobial technology research, photoactive advancements have concentrated on three pivotal modalities: photodynamic therapy (PDT), photothermal therapy (PTT), and photoinduced gas therapy [147, 148]. These modalities harness light as a noninvasive stimulant, offering immediate application and precise delivery capabilities. Photoactive antimicrobial materials are particularly favored for their ability to induce bacterial resistance and their efficacy against drug-resistant strains through unique sterilization mechanisms [149]. In recent years, photoactive materials have shown significant potential in the treatment of HP, leveraging their spatiotemporal controllability and lower resistance risk [128]. Compared with traditional therapies, PTT and PDT are less likely to induce drug resistance mutations, and through targeted light delivery, they can effectively target HP-infected areas while preserving the gut microbiota balance (Fig. 7) [89, 93, 96].

In the field of PTT, metal nanomaterials have garnered significant interest because of their exceptional NIR absorption capacity [147–149]. These materials efficiently convert light energy into thermal energy, presenting a novel PTT approach. This method disrupts bacterial survival by increasing local temperatures, resulting in potent antimicrobial effects, especially against drug-resistant bacteria [147–149]. Metal nanomaterials also play crucial roles in drug delivery and tissue engineering because of their biocompatibility, safety, and tunable optical properties [148, 149, 157]. Gold nanostars (GNSs), in particular, show superior potential in PTT because of their high NIR absorption and scattering and the presence of"hot spots"and sharp edges that facilitate efficient heat generation. The surfactant-free synthesis of GNSs enhances their biosafety [89]. Zhi et al. developed a thermosensitive GNS-based photothermal nanoprobe, GNS@Ab [89], which, under NIR irradiation, can increase the temperature in gastric infection areas, effectively eradicating drug-resistant HP (Fig. 7A). GNS@Ab can specifically bind to HP on the gastric mucosa and is safely excreted within 7 days after oral administration without disrupting the gut microbiota balance [89].

PDT, as a nonantibiotic strategy, uses specific laser wavelengths to activate photosensitizers (PSs), generating ROS that oxidize and damage bacteria and biofilms, achieving antimicrobial effects. PDT offers the advantage of eliminating bacteria and biofilms without inducing resistance, even with repeated applications [147–149]. Common PSs used in PDT include porphyrins and their derivatives, which efficiently produce singlet oxygen (^1^O_2_). Chlorine e6 (Ce6), an effective PS, is widely used in light-triggered delivery systems [146]. However, some PSs may exhibit cytotoxicity; hence, there is a need to develop HP-targeted PSs to minimize phototoxicity to normal cells. Wong and colleagues developed a novel zinc peroxide-based liposome nanoparticle, ZnO_2_-Ce6@lipo, capable of releasing Zn^2+^ and H_2_O_2_ in the acidic stomach environment, enhancing Ce6 efficacy and alleviating oxygen demand for photodynamic therapy (Fig. 7B) [96]. Under blue light, Ce6 generates 1 O_2_, and H_2_O_2_ photolysis produces hydroxyl radicals (•OH), forming a potent ROS reservoir that disrupts bacterial cell walls and facilitates Zn^2+^ entry into bacterial cells, effectively eradicating HP while safeguarding the gut microbiota [96].

The combination of PDT and PTT can overcome the limitations of single antimicrobial strategies, achieving a synergistic effect that surpasses the efficacy of individual treatments and effectively eradicates HP while preserving the gut microbiota balance [93, 142, 149]. This combined treatment reduces resistance risk, offers precise targeting, improves drug delivery, enhances antimicrobial capacity, reduces the inflammatory response, and modulates the immune response. Qiao et al. developed a near-infrared photosensitizer, T780 T, capable of achieving synergistic PTT and PDT effects [93]. By conjugating with a positively charged guanidine (Gu) group, T780 T-Gu enhances penetration and binding to negatively charged HPs through electrostatic interactions. Under NIR laser irradiation, T780 T-Gu converts light energy into heat and ROS, leading to cell membrane rupture and protein denaturation. Although T780 T-Gu shows significant antimicrobial and antibiofilm activity against HP in vitro, its in vivo performance is not satisfactory. Qiao et al. further combined the anionic biosurfactant rhamnolipid (RL) with T780 T-Gu to form RLs@T780 TG, improving mucus penetration and facilitating intelligent delivery and photothermal therapy upon light stimulation (Fig. 7C) [93]. RLs also inhibit biofilm formation by reducing bacterial adhesion.

Huang et al. developed a multifunctional oral nanomedicine, RHL@BP/ISL, utilizing a rhamnolipid-assisted black phosphorus nanocomposite, RHL@BP, to effectively deliver isorenieratene (ISL) [94]. ISL has excellent in vitro antimicrobial activity against HP, but its in vivo effect is limited. Black phosphorus (BP), an effective PTT-PDT agent, enhances the ability to combat HPs under NIR irradiation. The acid sensitivity of BP and the photothermal instability of RHL promote controlled ISL release combined with NIR irradiation to produce a PDT/PTT effect, demonstrating efficient anti-HP activity [94].

In addition, biocompatible materials based on low-virulence HP have been developed for macrophage reprogramming and immune modulation [150]. Zeng et al. [150] combined low-virulence type II HP with the photosensitizer Ce6 to create an HP-based engineered PDT system for treating gastric cancer. HP carrying Ce6 accumulates at the tumor site, and upon laser activation, PDT is initiated, triggering a burst of ROS. ROS not only combat the tumor but also degrade HP. The pathogen-associated molecular patterns (PAMPs) and antigens released after HP degradation can activate the cGAS-STING pathway and synergistically induce the polarization of macrophages towards the M1 phenotype in conjunction with ROS. This work provides an inspiring strategy for using HP as a tool to reshape the anticancer and antibacterial immune microenvironment, offering new insights for subsequent HP-based therapies [150].

The integrative therapeutic approach, which combines PDT with PTT, offers a dual advantage: it potentiates antimicrobial efficacy while concurrently preserving the equilibrium of the gut microbiota [28, 93]. PDT capitalizes on photosensitizers (PSs) to induce ROS, thereby neutralizing pathogenic bacteria, while PTT leverages the heat emitted by photothermal agents to augment therapeutic efficacy [89, 96]. This collaborative mechanism not only refines treatment results but also facilitates drug permeation through the gastric mucosa, ensuring profound penetration and comprehensive bacterial extermination. Moreover, this composite strategy exerts immunomodulatory and anti-inflammatory influences, which mitigates the necessity for extensive antibiotic use and concurrently diminishes the specter of antimicrobial resistance. Nonetheless, the disparate photophysical attributes inherent to PDT and PTT generally necessitate the employment of distinct laser sources, thereby complicating the treatment regimen. The innovation of a unifying molecular entity endowed with both PTT and PDT functionalities presents a promising avenue to streamline therapy and increase the precision of targeting [28, 32].

### Sonodynamic therapy (SDT)

Sonodynamic therapy (SDT) has emerged as an innovative therapeutic modality that leverages the synergistic interaction between ultrasound and sonosensitizers to achieve potent therapeutic effects [151]. The efficacy of SDT is largely attributed to the remarkable penetrative ability of ultrasound, which can easily traverse more than 10 cm of soft tissue, establishing its significance in the medical field. Owing to its noninvasive nature and profound tissue penetration, ultrasound is considered an exemplary external stimulus for medical applications [151–153].

The combination of ultrasound with sonosensitizers is particularly efficacious, as it can promote the generation of ROS at elevated concentrations. These ROS are inherently lethal to a diverse array of bacteria, yet they do not engender bacterial resistance [88, 98]. Furthermore, the precise targeting of ultrasonic energy to the afflicted region facilitates the activation and subsequent release of ROS from sonosensitizers. Consequently, SDT is adept at specifically addressing HPs within the gastric environment while simultaneously safeguarding the equilibrium of the intestinal flora without causing any disruption [96–98].

Sensitizers, as the core components of SDT, can generate ROS under the action of ultrasound. In the literature, sensitizers are mainly divided into two major categories: small organic molecule sensitizers and inorganic sensitizers. Recent research has shown that certain small-molecule sensitizers, such as indocyanine green (ICG), hematoporphyrin monomethyl ether (HMME), and verteporfin (Ver), have great potential for application [98]. For example, Wang et al. successfully prepared HPAb-LiP-ICG by encapsulating the sensitizer ICG in liposomes conjugated with HP monoclonal antibodies. This material can accurately identify and target HP through antigen‒antibody reactions and effectively eliminate HP by activating ICG with ultrasound to produce 1O_2_ while showing low toxicity to healthy tissues [98].

**Fig. 8:**
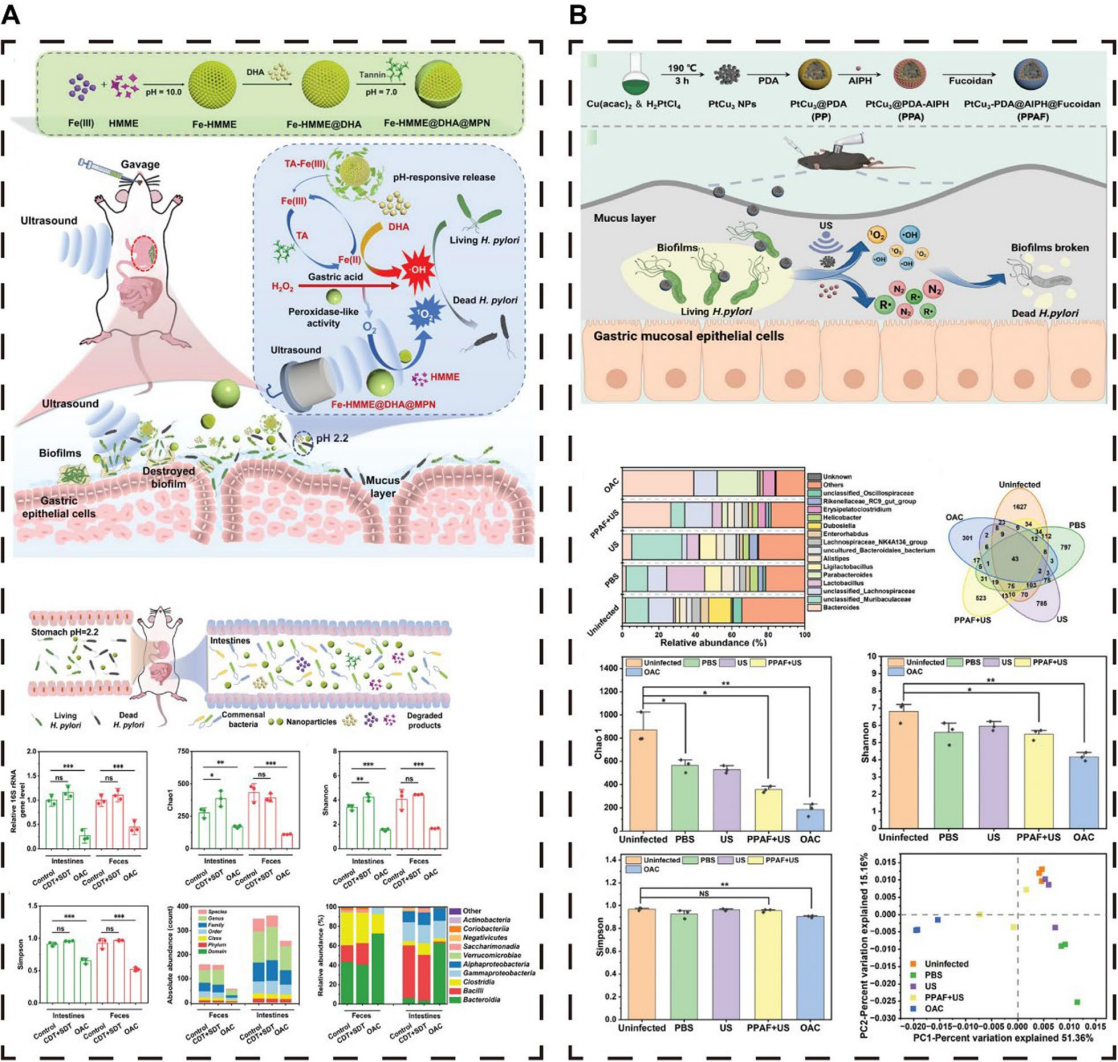
Biomaterial-mediated SDT resists HP and protects the intestinal microecology. **A** Multifunctional nanoenzymes combined with SDT and CDT produce 1O_2_ and ·OH. **B** Multifunctional nanoparticles combined with SDT and aeratotherapy produce 1O_2_, R· and N_2_. **A **Reproduced with permission from ref, [98] © Yu J (2023). B Reproduced with permission from ref, [88] © Fan J (2024)

Yu et al. developed a metal‒polyphenolic network (MPN) shell composed of iron and tannic acid and a mesoporous metal‒organic nanostructure Fe‒HMME (HMME, a sonosensitizer) core loaded with dihydroartemisinin (DHA), known as Fe‒HMME@DHA@MPN (Fig. 8A) [88]. In the acidic gastric environment, Fe-HMME and HMME dissociated from Fe-HMME@DHA@MPN, generating the 1O_2_ required for SDT under the action of ultrasound. Moreover, the dissociated porphyrin derivative (TA) can reduce Fe(III) to Fe(II), and Fe(II) can catalyze the reaction of hydrogen peroxide (H_2_O_2_) in the biofilm microenvironment of HP infection, resulting in the production of hydroxyl radicals (•OH) via chemodynamic therapy (CDT). Additionally, the oxygen generated by peroxidase catalysis helps alleviate the impact of hypoxic environments and further enhances the efficacy of SDT in eradicating HP. This design ingeniously utilizes the sonodynamic effect of ultrasound treatment, enabling the nanoparticles to produce more singlet oxygen than the traditional sonosensitizer HMME does, effectively eliminating multidrug-resistant HP and clearing biofilms. Notably, compared with conventional triple or quadruple therapies, the Fe-HMME@DHA@MPN nanogenerator has a negligible effect on the normal gut microbiota, thereby reducing the risk of side effects during treatment [88].

Liu et al. [97] developed a nanomaterial named Ver-PLGA@Lecithin, which features a precoated lecithin layer on its surface and is encapsulated with the sonosensitizer verteporfin in its core. This material, when exposed to ultrasound at an intensity of 0.5 W/cm^2^ for 10 min, effectively generates ROS, leading to the successful eradication of HP within the stomach. Compared with traditional antibiotic therapies, sonodynamic therapy has a minimal effect on the gut microbiota. Notably, it results in an increase in Lactobacillus, a common bacterium found in yogurt and probiotic products, which is the only significant alteration observed in the composition of the gut microbiota [97].

Inorganic nanosonosensitizers offer unique advantages over organic molecular sonosensitizers, such as optimized acoustic power performance through size and morphological adjustments, resistance to photobleaching, and the ability to serve as nucleation points for microbubble formation during ultrasonic cavitation. Gan et al. [88] fabricated a sonodynamic nanocomposite material, PtCu_3_-PDA@AIPH@Fucoidan (PPAF), which consists of the polydopamine-modified inorganic sonosensitizer PtCu3, a compound capable of generating alkyl radicals (R•), and fucoidan (Fig. 8B). PPAF has demonstrated the ability to penetrate the mucus barrier and target HP, disrupt the biofilm structure, and significantly enhance its bactericidal efficiency. In vitro experiments have shown that PPAF exhibits outstanding sonodynamic properties under ultrasonication, generating a substantial amount of ROS. Under ultrasonic stimulation, AIPH can release non-oxygen-dependent radicals (R•), which enhances the efficacy of SDT. Moreover, AIPH can also release N_2_, which further enhances the SDT effect of PPAF nanoparticles and aids in penetrating the gastric mucus layer and disrupting the biofilm structure. This leads to improved binding efficiency with biofilm bacteria, achieving the complete eradication of HP. In vivo experiments further confirmed the significant antibacterial effect of PPAF under ultrasonic stimulation. Compared with antibiotic treatment, PPAF has a lesser effect on the gastrointestinal microbiota, demonstrating its great potential as an alternative therapeutic approach [88].

SDT, recognized for its remarkable ability to penetrate and noninvasive characteristics, has become a preferred stimulus for therapeutic use [151–153]. When activated by ultrasound, sonosensitizers such as ICG are able to produce ROS, which can be precisely targeted to HPs by encapsulating them within carriers such as liposomes or nanoparticles. Compared with their inorganic counterparts, organic molecular sonosensitizers are superior in terms of biocompatibility and chemical stability; however, they offer unique advantages in terms of sonodynamic efficiency. SDT is an antibiotic-free antimicrobial approach that not only aids in reducing the development of antibiotic resistance but also minimizes potential disturbances to the gut microbiota. This study provides a new avenue for treating HP infections. Future studies should concentrate on refining sonosensitizers, evaluating the safety of the treatment, and monitoring its long-term effectiveness. Moreover, investigating how to seamlessly incorporate SDT into current clinical protocols is essential [28, 29].

### Magnetic hyperthermia

Magnetic therapy represents an innovative medical strategy that leverages the warmth effect of magnetic materials to precisely target cell membranes, creating localized"hot spots"that effectively disrupt the structure of HP [99, 154, 155]. This magnetothermal stimulation technique, which is characterized by its lack of penetration depth limitations, offers a minimally invasive and attractive physical method for regulating heat shock protein 70 (HSP70) within the body [99, 156]. Following infection, HP abnormally reduces the expression of HSP70 in gastric epithelial cells, thus promoting the progression of chronic infection. Studies suggest that inducing the expression of HSP70 could be a potential therapeutic strategy for protecting the gastric mucosa from HP damage [99, 156].

Superparamagnetic iron oxide nanoparticles (SPIONs), a traditional type of magnetic nanoheater, are widely used in various fields [154, 155]. However, they face the challenge of gastric acid corrosion during gastric cancer treatment. Gastric acid may cause the decomposition of SPIONs, releasing potentially toxic substances, which limits their direct application in gastric treatment. Therefore, the development of new magnetic nanomaterials that can adapt to the acidic environment of the stomach is a critical direction for future research [154].

**Fig. 9:**
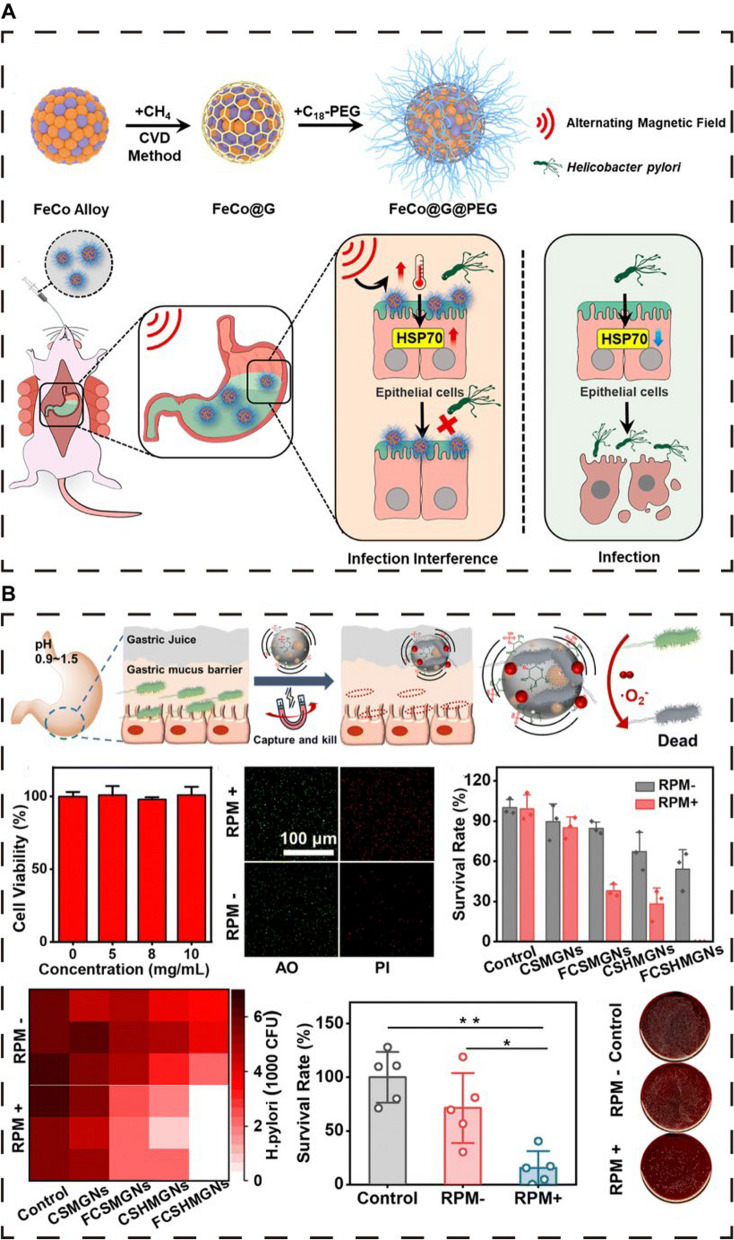
Biomaterial-mediated MHTs resist HP and protect the intestinal microecology. **A** Multifunctional nanosphere-mediated MHT. **B** Magnetic nanorobots jointly mediate MHT and CDT. **A **Reproduced with permission from ref, [99] © Xia X (2022). B Reproduced with permission from ref, [100] © Cai X (2023)

Xia et al. encapsulated an iron‒cobalt alloy with graphene layers to successfully develop a novel gastric nanoheater, FeCo@G (Fig. 9A) [99]. This nanocomposite material demonstrates exceptional stability and high specific energy loss efficiency in acidic environments, providing effective magnetothermal therapy for the stomach. This treatment promoted the expression of HSP70 in gastric epithelial cells, increasing their resistance to HP infection. The FeCo@G nanocomposite has shown significant inhibitory effects on HP in vivo and good biocompatibility, with 95% of the nanocomposite safely excreted from the gastrointestinal tract of mice within 12 h. The development of this nanoheater allows magnetothermal therapy to be applied in the complex environment of the stomach, offering a new strategy for the treatment of HP [99].

HSP70, an important cellular protective factor, plays an auxiliary role in the development of bacterial vaccines, and its key role during HP infection has been extensively studied [28, 99]. HSP70 can respond to a variety of stress conditions, including increased temperature, oxidative stress, radiation, chemical exposure, and nutrient deficiency. Among these stress factors, an increase in temperature is a relatively mild and side effect-free method of activation. The development of nanoheaters that can operate stably in acidic environments to regulate HSP70 levels in the digestive tract effectively disrupts the infection process of HP in the body, providing new tools for exploring new antimicrobial strategies [28, 99].

Cai et al. [100] developed engineered microsweepers featuring a single-iron-atom catalyst to locate and neutralize HP infections (Fig. 9B). These microsweepers, under active guidance, execute extensive, wall-clinging oscillatory movement, increasing their contact with HP and subsequently suppressing bacteria by generating acid-responsive reactive oxygen species [100].

However, there are differences in the effects of magnetothermal therapy on bacteria and human cells, and how to precisely control the local magnetothermal effect and ensure that it is within the therapeutic range is a problem that requires further research. The precise control of magnetothermal therapy to ensure its safety and efficacy in the treatment of HP infections remains a significant challenge and an area ripe for future investigation [155, 156].

In summary, multimodal therapies, including phototherapy, sonodynamic therapy, and magnetic hyperthermia, have shown remarkable potential in eradicating HP while safeguarding the intestinal microbiome [28, 93, 98, 99]. These advanced approaches leverage stimuli-responsive biomaterials to achieve precise targeting and controlled release of antimicrobial agents. By integrating the principles of HIM and the 3R concept, these therapies not only eliminate HP but also promote mucosal healing and maintain microbial balance. The"Remove"phase targets direct eradication of HP, while"Remodel"focuses on reshaping the immune microenvironment by modulating macrophages and T cells to clear pathogens."Repair"emphasizes gastric mucosa restoration and gut microbiota protection. Future research should focus on optimizing these materials and validating their efficacy through rigorous in vivo and clinical studies to fully realize their potential in HP management.

## Conclusion and future perspective

HP infection remains a significant global health challenge, with traditional antibiotic therapies facing increasing resistance rates and adverse effects on the intestinal microbiota [157–161]. This review has highlighted the potential of biomaterial-based multimodal therapies guided by the principles of HIM and the 3R concept to address these challenges effectively. By integrating advanced biomaterials with targeted delivery systems and non-invasive stimuli-responsive therapies, we can achieve precise eradication of HP while preserving the integrity of the gut microbiome.

The HIM approach emphasizes a patient-centered strategy that considers the overall health impact of treatments, beyond merely targeting the pathogen [26–28]. This holistic perspective is crucial in developing therapies that not only eliminate HP but also promote mucosal healing, modulate inflammation, and maintain microbial balance [162–165]. The 3R concept further refines this approach by focusing on the removal of pathogens, remodeling of the gastric microenvironment, and repair of the gastric mucosa. Biomaterials, such as nanoparticles, hydrogels, and microspheres, have shown remarkable potential in realizing these goals. For instance, pH-responsive nanoparticles can selectively release antimicrobial agents in the acidic gastric environment, minimizing off-target effects on the intestinal flora [33]. Similarly, hydrogels can provide a controlled release of therapeutic agents, enhancing their efficacy and reducing systemic toxicity [34, 35].

However, translating these promising preclinical findings into clinical practice requires careful consideration of several factors [166–168]. The differences between preclinical animal models and human patients are significant, particularly in terms of pharmacokinetics, pharmacodynamics, and potential systemic toxicity [166, 167]. While small animal models, such as mice, have been invaluable in initial studies, they may not fully replicate the complexity of human physiology and pathology [166, 167]. Therefore, exploring other animal models, such as larger mammals (*e.g.*, pigs or non-human primates), could provide more relevant insights into the efficacy and safety of these biomaterial-based therapies. These models can better mimic the human gastrointestinal tract's anatomy and physiology, offering a more accurate assessment of the therapeutic potential and potential adverse effects [166–168].

Moreover, the dosing regimens and administration routes need to be optimized to ensure effective delivery of therapeutic agents to the target site while minimizing systemic exposure [170–173]. The potential for systemic toxicity, especially with the use of metal-based nanoparticles, must be thoroughly evaluated. Long-term studies are necessary to understand the chronic effects of these materials on human health, including potential neurotoxicity and immunological responses [170–173].

**Fig. 10:**
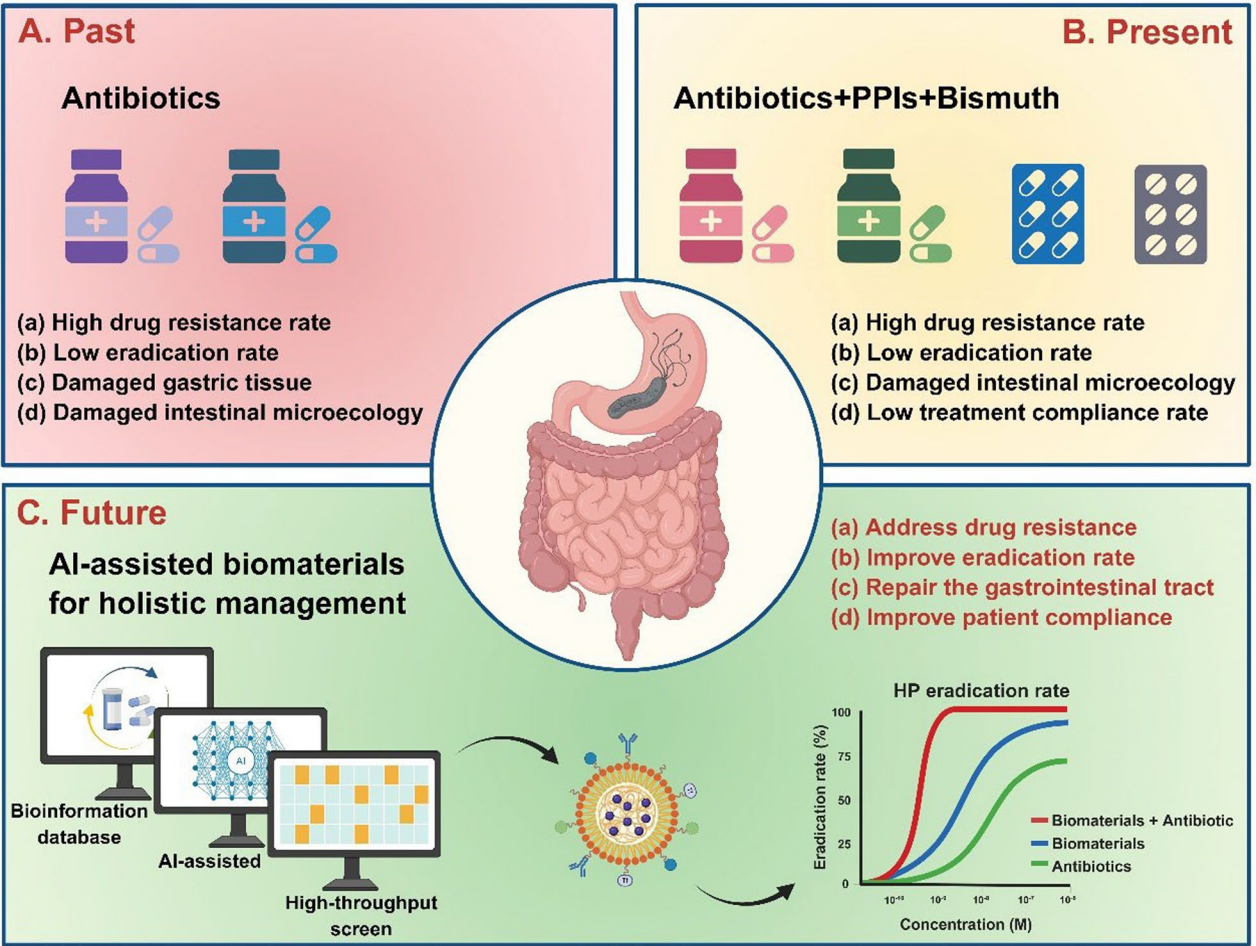
The past, present and future of HP therapy. The transition from past antibiotic-centric approaches to present combinations of antibiotics, PPIs, and bismuth faces ongoing challenges such as drug resistance and microbiome disruption. Future advancements will integrate AI-assisted biomaterials and databases to develop targeted, multimodal therapies. These will not only address drug resistance but also enhance eradication rates and repair gastrointestinal tracts, improving patient compliance. This holistic strategy promises safer, more effective treatments aligned with HIM principles and the 3R concept. (Created with BioRender.com)

Future research should focus on developing sophisticated biomaterials integrating multiple therapeutic modalities, such as phototherapy, sonodynamic therapy, and magnetic hyperthermia, with targeted drug delivery systems to enhance precision and efficacy in HP eradication [28, 175]. Leveraging advanced technologies like CRISPR-Cas9 for gene editing could address antibiotic resistance by targeting resistance genes in HP. Additionally, incorporating biomaterials and drug information databases with AI-assisted design and high-throughput screening will accelerate the discovery and optimization of novel therapeutics [176–181]. This approach, as depicted in Fig. 10, aligns with HIM principles and the 3R concept, offering a promising direction for managing HP infections. It emphasizes broader health implications and targeted, multifaceted strategies to develop more effective and safer treatments, ultimately improving patient outcomes and reducing the global burden of HP-related diseases.

## Supplementary Information

Additional file 1.

## Data Availability

The data that support the findings of this study are available from the corresponding author upon reasonable request. No datasets were generated or analysed during the current study.
