# Supplementary material for: Biomaterials mediated 3R (remove-remodel-repair) strategy: holistic management of Helicobacter pylori infection
Source: J Nanobiotechnology. 2025 Jul 1;23:475. doi: 10.1186/s12951-025-03455-2 (PMC12211381; doi:10.1186/s12951-025-03455-2)
Supplement: Supplementary file 1 — Additional file 1. [file 12951_2025_3455_MOESM1_ESM.docx]

***Biomaterials Mediated 3R (remove-remodel-repair) Strategy: Holistic Management of Helicobacter pylori Infection***

Tinglin Zhang ^1,2†^, Yating Zheng ^1,7†^, Tielou Chen ^4†^, Yuankai Gu ^5†^, Yingli Gong ^1,6^, Dewei Wang ^8^, Zhaoshen Li ^1,2,3*^, Yiqi Du ^1,2,3*^, Li Zhang ^1,2*^, Jie Gao ^1,2*^

^†^ Tinglin Zhang, Yating Zheng, Tielou Chen, Yuankai Gu have contributed equally to this work.

^*^ Correspondence:

Jie Gao

[gaojiehighclea@smmu.edu.cn](mailto:gaojiehighclea@smmu.edu.cn)

Li Zhang

[lizhangpaper@163.com](mailto:lizhangpaper@163.com)

Yiqi Du

[duyiqi@hotmail.com](mailto:duyiqi@hotmail.com)

Zhaoshen Li

[zhsl@vip.163.com](mailto:zhsl@vip.163.com)

Full list of author information is available at the end of the article.

**Author details**

^1^ Changhai Clinical Research Unit, Shanghai Changhai Hospital, Naval Medical University, Shanghai 200433, China. ^2^ Shanghai Key Laboratory of Nautical Medicine and Translation of Drugs and Medical Devices, Shanghai 200433, China. ^3^ Department of Gastroenterology, Shanghai Changhai Hospital, Naval Medical University, Shanghai 200433, China. ^4^ Department of Stomatology, Shanghai Changhai Hospital, Naval Medical University, Shanghai 200433, China. ^5^ Department of Neurology, Shanghai Changzheng Hospital, Naval Medical University, Shanghai 200003, China. ^6^ College of Life Science, Mudanjiang Medical University, Mudanjiang 157011, China. ^7^ Yangzhou Branch of Jiangsu Provincial Corps of Chinese People’s Armed Police Force, Jiangsu 225007, China. ^8^ College of Science, University of Shanghai for Science and Technology, Shanghai 200433, China.

**
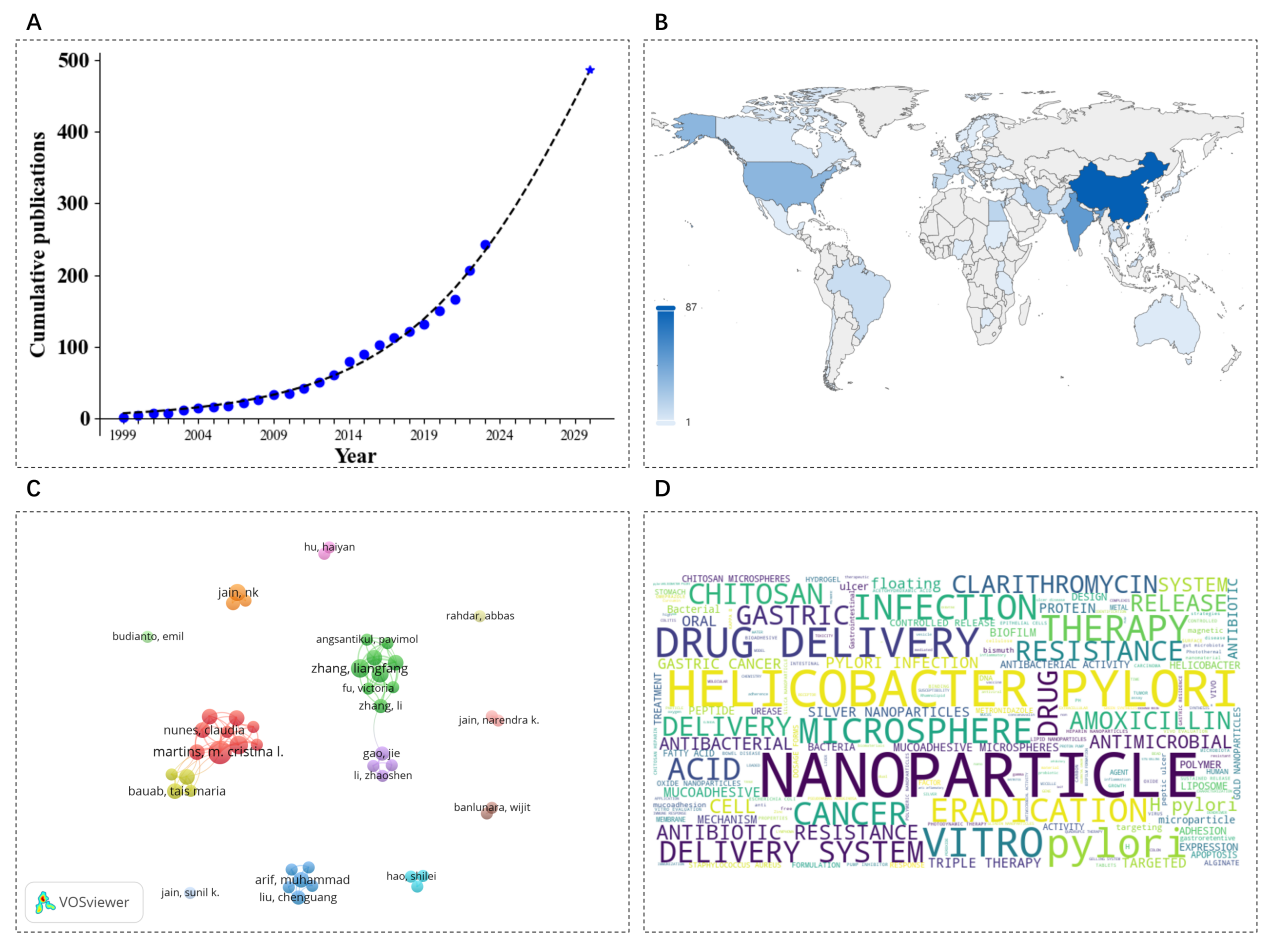
**

**Figure S1. A)** Logistic growth curve. **B)** Global perspective of publication volume from the world map. **C)** uses VOSviewer to visualize the cooperation between authors, which indicates the prosperous view of this field. **D)** Outbreak citation analysis. the most popular journals and the most influential journals, membership chart.

**Table S1. Top 10 productive countries**

| Country | N | % | Total citations | Average citations | H-index |
| --- | --- | --- | --- | --- | --- |
| CHINA | 87 | 22.48 | 1716 | 19.72 | 23 |
| INDIA | 51 | 13.18 | 1383 | 27.12 | 24 |
| USA | 34 | 8.79 | 2606 | 76.65 | 20 |
| IRAN | 24 | 6.2 | 280 | 11.67 | 9 |
| PORTUGAL | 17 | 4.39 | 425 | 25 | 11 |
| EGYPT | 15 | 3.88 | 162 | 10.8 | 8 |
| FRANCE | 12 | 3.1 | 1349 | 112.42 | 10 |
| ITALY | 11 | 2.84 | 828 | 75.27 | 9 |
| ARABIA | 11 | 2.84 | 178 | 16.18 | 5 |
| ENGLAND | 10 | 2.58 | 308 | 30.8 | 8 |

**Table S2. Top 10 productive Affiliations**

| Affiliation | N | % | Total citations | Average citations | H-index |
| --- | --- | --- | --- | --- | --- |
| UNIVERSIDADE DO PORTO | 16 | 2.39 | 423 | 26.44 | 11 |
| EGYPTIAN KNOWLEDGE BANK (EKB) | 15 | 2.24 | 162 | 10.8 | 8 |
| DR. HARI SINGH GOUR UNIVERSITY | 11 | 1.64 | 495 | 45 | 10 |
| I3S - INSTITUTO DE INVESTIGACAO E INOVACAO EM SAUDE | 11 | 1.64 | 198 | 18 | 7 |
| CHINESE ACADEMY OF SCIENCES | 9 | 1.34 | 380 | 42.22 | 7 |
| OCEAN UNIVERSITY OF CHINA | 9 | 1.34 | 150 | 16.67 | 7 |
| UNIVERSITY OF CALIFORNIA SYSTEM | 8 | 1.19 | 1083 | 135.38 | 8 |
| SUN YAT SEN UNIVERSITY | 8 | 1.19 | 195 | 24.38 | 7 |
| KING SAUD UNIVERSITY | 6 | 0.9 | 55 | 9.17 | 4 |
| TEHRAN UNIVERSITY OF MEDICAL SCIENCES | 6 | 0.9 | 26 | 4.33 | 3 |

**Table S3. Top 10 Productive Authors**

| Affiliation | N | % | Total citations | Average citations | H-index |
| --- | --- | --- | --- | --- | --- |
| MARTINS, M. CRISTINA | 12 | 0.7 | 334 | 27.83 | 8 |
| ZHANG, LI | 11 | 0.64 | 1137 | 103.36 | 10 |
| JAIN, S | 9 | 0.52 | 314 | 34.89 | 9 |
| ZHANG, LIANG | 8 | 0.46 | 1021 | 127.62 | 7 |
| ZHANG, LIANGFANG | 7 | 0.41 | 1021 | 145.86 | 7 |
| GONCALVES, INES C. | 7 | 0.41 | 228 | 32.57 | 6 |
| THAMPHIWATANA, SORACHA | 6 | 0.35 | 886 | 147.67 | 6 |
| JAIN, NK | 6 | 0.35 | 289 | 48.17 | 6 |
| OBONYO, MARYGORRET | 5 | 0.29 | 536 | 107.2 | 5 |
| GAO, WEIWEI | 5 | 0.29 | 482 | 96.4 | 5 |
| REIS, SALETTE | 5 | 0.29 | 174 | 34.8 | 4 |
